# Invasive listeriosis outbreaks and salmon products: a genomic, epidemiological study

**DOI:** 10.1080/22221751.2022.2063075

**Published:** 2022-05-23

**Authors:** Raskit Lachmann, Sven Halbedel, Stefanie Lüth, Alexandra Holzer, Marlen Adler, Ariane Pietzka, Sascha Al Dahouk, Klaus Stark, Antje Flieger, Sylvia Kleta, Hendrik Wilking

**Affiliations:** a FG35 Division of Gastrointestinal Infections, Zoonoses and Tropical Infections, Robert Koch Institute, Berlin, Germany; b FG11 Division of Enteropathogenic Bacteria and Legionella, Consultant Laboratory for Listeria, Robert Koch Institute, Wernigerode, Germany; c German Federal Institute for Risk Assessment, National Reference Laboratory for Listeria Monocytogenes, Berlin, Germany; d Austrian Agency for Health and Food Safety, Graz, Austria

**Keywords:** Listeriosis, salmon products, foodborne outbreaks, public health, molecular surveillance

## Abstract

Invasive listeriosis, caused by *Listeria (L.) monocytogenes*, is a severe foodborne infection, especially for immunocompromised individuals. The aim of our investigation was the identification and analysis of listeriosis outbreaks in Germany with smoked and graved salmon products as the most likely source of infection using whole-genome sequencing (WGS) and patient interviews. In a national surveillance programme, WGS was used for subtyping and core genome multi locus sequence typing (cgMLST) for cluster detection of *L. monocytogenes* isolates from listeriosis cases as well as food and environmental samples in Germany. Patient interviews were conducted to complement the molecular typing. We identified 22 independent listeriosis outbreaks occurring between 2010 and 2021 that were most likely associated with the consumption of smoked and graved salmon products. In Germany, 228 cases were identified, of 50 deaths (22%) reported 17 were confirmed to have died from listeriosis. Many of these 22 outbreaks were cross-border outbreaks with further cases in other countries. This report shows that smoked and graved salmon products contaminated with *L. monocytogenes* pose a serious risk for listeriosis infection in Germany. Interdisciplinary efforts including WGS and epidemiological investigations were essential to identifying the source of infection. Uncooked salmon products are high-risk foods frequently contaminated with *L. monocytogenes*. In order to minimize the risk of infection for consumers, food producers need to improve hygiene measures and reduce the entry of pathogens into food processing. Furthermore, susceptible individuals should be better informed of the risk of acquiring listeriosis from consuming smoked and graved salmon products.

## Introduction

Invasive listeriosis is a severe, foodborne infection caused by *Listeria (L.) monocytogenes*, with high hospitalization and case fatality rates compared to other gastrointestinal bacterial pathogens. Invasive listeriosis is most commonly seen in the elderly, immunocompromised patients, pregnant women, and newborns and can cause sepsis, meningitis, encephalitis, neonatal infections, stillbirths, or abortions [1].

In Germany, analyses of surveillance data revealed an average incidence rate of 0.69 per 100,000 population with a case fatality of 13% in non-pregnancy-associated cases. A steady increase in case numbers was observed between 2011 and 2017, followed by a slight decrease in 2019 and 2020 [1]. Altogether, 91% of the listeriosis cases were non-pregnancy-associated and among these, there was a preponderance of men (60%).

In 2018, after comprehensive method comparison/validation, real-time whole-genome sequencing (WGS)-based typing of *L. monocytogenes* isolates from clinical cases, food and production environments replaced pulsed-field gel electrophoresis typing for routine detection and clarification of outbreaks in Germany in the national Listeria surveillance programme [2–6]. The identification of outbreak vehicles is difficult due to the relatively low number of cases available for epidemiological investigations and the challenge of obtaining reliable consumption histories after long incubation periods from critically ill patients [7]. Listeriosis outbreaks may affect several countries and last for several years, making it difficult to link affected patients without the use of WGS [3,8,9]. Systematic WGS-based typing of *L. monocytogenes* isolates from food products enables the identification of outbreak vehicles [2,10–13]. Using WGS-based typing, several large nationwide outbreaks were successfully detected, investigated, and stopped in Germany [3,4,10]. Here, we describe the identification and investigation of 22 outbreaks with 228 listeriosis cases occurring from 2010 to 2021 with smoked or graved salmon products as the most likely source of infection. We focus on salmon-associated outbreaks to illustrate the significant and preventable public health problem of listeriosis due to contaminated smoked or graved salmon products.

## Materials and methods

*L. monocytogenes* strains isolated from listeriosis patients (referred to as clinical isolates) were collected from primary diagnostic labs in Germany grown on Brain Heart Infusion (BHI) agar or BHI broth at 37°C [4,14]. Strains were deposited in the in-house strain collection of the consultant laboratory for *Listeria* (consultant laboratories are national laboratories in Germany, responsible for laboratory surveillance of important pathogens).

DNA was isolated by mechanical disruption using glass beads in a TissueLyser II bead mill (Qiagen, Hilden, Germany) and quantified using a Qubit dsDNA BR (or HS) Assay kit and Qubit fluorometers (Invitrogen™, Carlsbad, CA, USA). Libraries were generated using the Nextera XT DNA Library Prep Kit (Illumina Inc., San Diego, CA, USA). Genome sequencing was performed on MiSeq, HiSeq, or NextSeq sequencers. Trimming of raw reads and contig assembly was executed in Ridom Seqsphere + using Velvet or Spades as an assembler. Sequencing coverage ranged between 21- and 176-fold (median 54-fold). Clinical isolates from 2014 and earlier were retrospectively sequenced and recognized as outbreak cases.

In this study, we included 166 *L. monocytogenes* isolates from fish and fish-processing environments (referred to as non-clinical isolates), sampled as part of official food control activities between 2011 and 2021. Isolates were grown on sheep blood agar at 37°C. The PulseNet protocol for Gram-positive bacteria was used for cell lysis (https://www.cdc.gov/pulsenet/pdf/pnl32-miseq-nextera-xt.pdf), followed by DNA extraction using the QIAmp DNA Mini Kit (Qiagen, Hilden, Germany) or the PureLinkTM Genomic DNA Mini Kit (Invitrogen™, Carlsbad, CA, USA). DNA was quantified using the QubitTM dsDNA BR Assay Kit with the QubitTM 2.0 Fluorometer (InvitrogenTM, Carlsbad, CA, USA). The sequencing library was prepared with the Illumina Nextera XT DNA Library Kit or the DNA Prep Kit (Illumina Inc., San Diego, CA, USA). Sequencing was performed in paired-end mode with 2 × 150 bp using the Illumina NextSeq 500 or with 2 × 300 bp using the Illumina MiSeq (Illumina Inc., San Diego, CA, USA). Trimming of sequencing raw reads, assembly, and overall quality assessment were performed using the pipeline AQUAMIS [15]. Accession numbers of isolate sequencing data are presented in Supplemental Table 1. Non-clinical isolates are deposited in the strain collection of the National Reference Laboratory for Listeria monocytogenes at the German Federal Institute for Risk Assessment (BfR).

Molecular serogroups, MLST sequence types (STs), and 1701 locus cgMLST complex types (CTs) were calculated in Ridom Seqsphere + by automated allele submission to the *L. monocytogenes* cgMLST server (http://www.cgmlst.org/ncs/schema/690488/) [16]. ST assignments were not possible for three isolates due to incomplete allele coverage (Supplemental Table 1). Minimum spanning trees were calculated in the “pairwise ignore missing values” mode and groups of isolates with ≤7 different alleles between neighbouring isolates were considered as genetically closely related cgMLST cluster. For the purpose of this analysis, outbreaks are defined as two or more patients for which the same source of infection has to be assumed since their clinical isolates belong to the same cgMLST cluster.

In 2019, a cgMLST cluster naming system for public health communication purposes was introduced using a combination of Greek letters and numbers to make listeriosis outbreaks more recognizable and distinguishable for stakeholders in surveillance and outbreak management. For this reason, this designation is mentioned together with the cgMLST in our analysis.

Listeriosis patients were defined as patients that were reported to public health authorities and thence to the Robert Koch Institute (RKI) with the onset of listeriosis since 2010, proven by the isolation of *L. monocytogenes* from normally sterile body fluids or in the context of birth from a normally non-sterile site. Outbreak cases were defined as listeriosis cases belonging to specific cgMLST clusters.

Following a diagnosis of listeriosis, patients were interviewed using a standardized questionnaire asking for their food consumption in the two weeks before symptom onset, their general food purchasing behaviours, and their medical history. The incubation period of 1 to 14 days was defined according to Goulet et al. [7].

Member states of the EU and other countries have been informed about these outbreaks via the Epidemic Intelligence Information System for Food and Waterborne Diseases (EPIS-FWD) of the European Center for Disease Prevention and Control (ECDC) and have been asked to provide information about genetically closely related cases in their countries.

The outbreak investigations and publication were executed according to the German Infection Protection Act as part of the mandate of the local public health agencies and RKI as a national public health institute.

## Results

**Figure 1.**
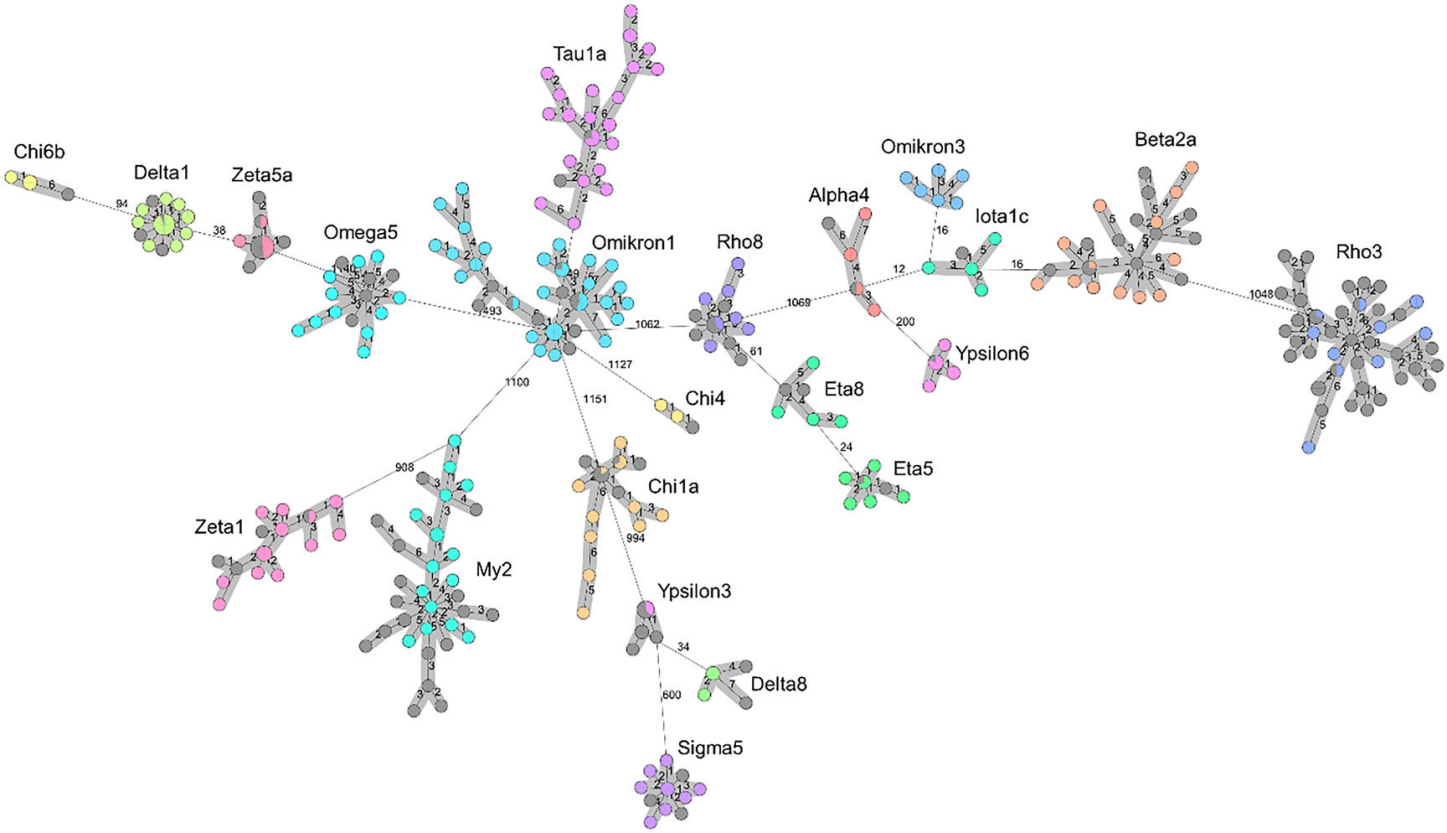
Minimum spanning tree showing 22 listeriosis clusters associated with salmon consumption. The tree was calculated using 1701 locus cgMLST data in the pairwise ignore missing values mode. Clinical isolates are shown in various colours, food isolates are shown in grey. Clusters consisting of isolates with ≤7 different alleles are highlighted by a grey background. Numbers indicate allelic distances between the single nodes of the tree.

Within each of the 22 listeriosis outbreaks identified using cgMLST, a close genetic relationship was found between the clinical *L. monocytogenes* isolates and non-clinical isolates from smoked or graved salmon products. The *L. monocytogenes* clones in the 22 outbreaks are generally not closely related to each other at the molecular level (Figure 1). In total, 31 cgMLST cluster types and 4 serovars were identified (Table 1). Many of the outbreaks were caused by molecular serogroup IIa clones (18/22; 81%), even though only appr. 40% of all human listeriosis cases in Germany were due to IIa strains in a previous analysis [2]. The genome sequences of clinical isolates within the individual outbreaks are closely related. Therefore, we assumed a common source of infection for the respective patients (Figure 1).

**Table 1.** Characteristics of 22 listeriosis outbreaks with salmon products as the most likely source in Germany, 2010–2021.

Cluster name^a^	Serovar	Cluster type^b^	Number of notified cases	Number of notified case by year					Number of notified cases by sex		Age in years			Number of deceased case		Number of pregnancy-associated cases	Number of clinical isolates	Number and year of non-clinical isolates	RASFF notification^c^	Cases outside of Germany
				2010–2017	2018	2019	2020	2021 (until 30.09.)	Male	Female	Minimum	Maximum	Median	Deceased	Deceased due to listeriosis					
Alpha4	IIa	1269	5	2	0	2	1	0	2	3	68	91	80	2	0	0	5	1x2016, 1x2017		yes
Beta2a	IIa	1247	12	3	1	0	2	6	8	4	58	86	79	2	1	0	13	1x2011, 1x2013, 3X2017, 1x2018, 2x2020, 7x2021	2016/1789	yes
Chi1a	IIa	2966, 5583	7	4	1	2	0	0	4	3	53	90	84	1	0	0	12	1x2012, 2x2014, 1x2015, 3x2016, 2x2017		yes
Chi4	IIa	4035	2	0	2	0	0	0	1	1	72	76	74	0	0	0	2	1x2016		
Chi6b	IVb	1738, 9071	5	0	0	5	0	0	2	3	60	93	81	1	0	0	5	1x2016		
Delta1	IVb	3530	20	19	1	0	0	0	5	15	24	94	77	2	2	0	22	1x2013, 3x2016,1x2018	2013/0854	yes
Delta8	IIa	4295	3	0	1	1	1	0	2	1	81	87	81	1	0	0	4	1x 2013, 1X2017		
Eta5	IIa	5488	7	2	0	3	2	0	3	4	53	93	80	2	0	0	7	1x2016, 1x2017	2019/4238	
Eta8	IIa	4230	3	0	0	1	1	1	2	1	63	83	73	0	0	0	4	2X2016		
Iota1c	IIa	73, 6756	5	2	1	1	1	0	1	4	68	83	77	2	1	0	5	1x2016		
My2	IIa	3242	17	5	0	7	4	1	10	7	24	91	74	5	1	1	19	1x2016, 1x2017, 3x2020, 14x2021		yes
Omega5	IIb	773, 1138	10	0	2	5	3	0	8	2	0	90	78	2	1	0	11	2x2016, 1x2018, 1x2020, 1x2021		yes
Omikron1	IIa	1128	37	16	0	5	14	2	22	15	13	90	78	9	4	0	42	3x2016, 4x2017, 4x2020, 1x2021	2019/4292	yes
Omikron3	IIa	2994, 4997	7	4	3	0	0	0	4	3	22	82	78	4	1	0	8		2017/1319 2017/1546	yes
Rho3	IIa	1690	8	5	3	0	0	0	5	3	56	92	75	1	0	0	11	2x2014, 27x2016, 10x2017, 2x2018, 1x2019, 4x2020	2016/1290	yes
Rho8	IIa	7559	9	0	1	0	7	1	4	5	0	92	82	2	0	1	9	2x2017, 1x2019, 4x2020, 3x2021		yes
Sigma5	IIa	5715	8	0	2	0	5	1	4	4	17	84	80	2	0	0	10	1x2016, 2X2020	2021/0065	
Tau1a	IIa	2198	24	10	11	2	1	0	15	9	35	91	79	7	3	0	30	1x2016, 2x2017		yes
Ypsilon3	IIa	5554	2	0	2	0	0	0	2	0	61	64	63	1	1	0	3	3x2016, 7x2017		
Ypsilon6	IIa	3732	7	0	0	5	1	1	3	4	63	85	77	0	0	0	8	1x2017		
Zeta1	IIa	40, 3991, 6406	19	16	0	0	2	1	11	8	2	96	76	3	2	0	19	1x2016, 3x2017		
Zeta5a	IVb	3386	11	1	8	2	0	0	5	6	29	99	66	1	0	2	10	13x2018		yes

^a^ In 2019, a cgMLST cluster naming system was introduced using a combination of Greek letters and numbers to make listeriosis outbreaks more recognizable and distinguishable for stakeholders.

^b^ Cluster Type according to Ruppitsch et al.

^c^ The Rapid Alert System for Food and Feed (RASFF) is a notification system operated by the European Commission to exchange information on identified hazards between Member States and covers food, food contact materials, and animal feed.

By cgMLST analysis, a total of 166 non-clinical isolates, 153 from fish products, and 13 from fish-processing plants were closely related to the 22 outbreak clusters (per outbreak 1 to 46, median four), using the 259 clinical isolates from listeriosis patients. The allelic difference (AD) between a non-clinical and a clinical isolate within an outbreak was between zero and 14 (Supplemental Table 2). Non-clinical isolates were collected over one to six years (median two) and in one to eight (median two) of the 16 German Federal States for each outbreak. For 20 outbreaks, closely related non-clinical isolates were only from food, in one outbreak, closely related isolates were also from the fish-associated processing environment. For one outbreak, no closely related isolates could be found in Germany. This cluster resulted from a cross-border outbreak and its association with salmon was assumed based on food isolates from Denmark [9,17].

The vast majority of non-clinical isolates came from salmon and salmon products (*n* = 138, 90% of the food isolates). For each of the outbreaks, at least one of the closely related food isolates originated from smoked or graved salmon. Another 15 non-clinical isolates, closely related to the outbreak clusters, too, were from various other fish products, e.g. smoked trout (*n* = 3), pickled herring salad (*n* = 3), or smoked halibut (n = 2).

From 2018 to 2020, clinical isolates were sequenced for 1085 of the 1873 notified listeriosis cases. Microbiological and epidemiological information on suspected food vehicles could be retrieved for 466 of these cases and 124 cases (27%) were likely caused by smoked or graved salmon products. RKI-internal names were assigned to the outbreaks and detailed information on these 22 outbreaks can be found in Table 1.

**Figure 2.**
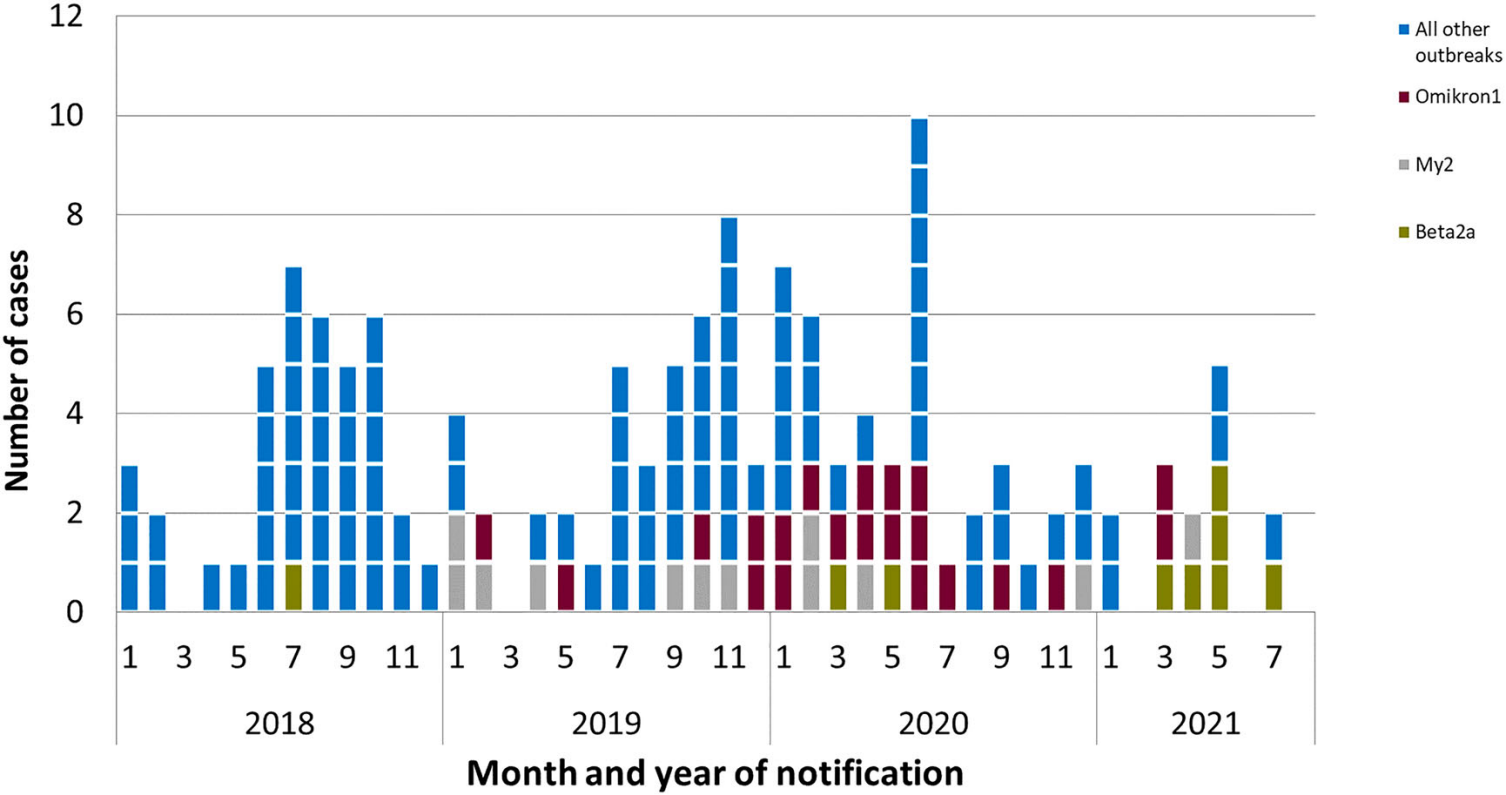
Outbreak cases by month and year of notification, Germany 2018–2021. Cases are shown since 2018 when all available clinical isolates where sequenced. Three large ongoing outbreaks (Beta2a in green, My2 in grey, and Omikron1 in brown) are presented individually, the other 19 outbreaks are shown in blue.

Two hundred and fifty-nine clinical isolates belonging to the 22 outbreaks were identified in the consultant laboratory between 2010 and September 2021. The isolates could be allocated to 228 listeriosis cases, which were notified and transmitted according to the Infection Protection Act (IfSG) (Table 1 and Figure 2). The patients acquired listeriosis between 2010 and 2021. The clusters were all geographically widespread in Germany and all federal states except for Saarland were affected. To our knowledge so far, more than 75 clinical isolates have been identified in 15 other countries that are genetically very closely related to isolates from 12 of the outbreaks described here. The ease of sharing isolate sequencing data between different countries facilitated international investigations of listeriosis outbreaks.

The number of cases in the outbreaks in Germany ranged from 2 to 37 cases (median of 8 cases). Fifty people were reported to the RKI as deceased, of which 17 were reported to have died directly or indirectly from listeriosis. Four pregnancy-associated cases were reported.

Interviews on food consumption and shopping behaviour were carried out with a total of 27 patients or relatives of patients from 13 outbreaks. Of these, 22 (81%) reported either consumption of salmon, smoked or graved salmon or smoked fish.

## Discussion

Smoked or graved salmon products were identified as the most likely source for 22 independent outbreaks in Germany since 2010. The presence of closely related *L. monocytogenes* isolates from food monitoring in Germany provides strong microbiological evidence that the sources for the outbreaks described here were smoked and/or graved salmon products [18]. The Omikron3 outbreak also affected Denmark and was linked independently to the consumption of cold-smoked salmon by a Danish investigation [17]. Importantly, isolates from salmon products and fish-processing environments identified in a Polish study also belong to the Alpha4, Beta2a, My2, and Omikron1 clusters [19]. Thus, the trade of contaminated salmon products between different European countries is associated with some of the German outbreaks.

Altogether, over 80% of interviewed patients in Germany recalled consumption of smoked salmon or smoked fish in the two weeks before the onset of disease. This is more than would be expected in the general population even though only a small proportion of patients could be interviewed. In a food consumption survey for outbreak investigations in Germany, 24% (95%-CI 18–30%) of healthy over 65-year-old respondents stated that they had consumed smoked or graved salmon in a period of two weeks prior to the survey [20]. These results from patient interviews provide convincing epidemiological evidence and support the causal relationship between listeriosis outbreaks and the consumption of salmon [18].

Before our analysis, smoked and graved salmon were already assessed as a risk product for listeriosis. Our data not only confirm this assessment for the first time with data from patients in Germany, it also shows the magnitude of the problem. From 2018 to 2020, in Germany 27% of all listeriosis cases with suspected food vehicles were likely caused by smoked or graved salmon products. Considering that for another 1407 listeriosis cases no food vehicle has been identified yet, it is reasonable to assume that many of these cases were also caused by smoked or graved salmon products. Therefore, we focused this analysis on smoked and graved salmon products as we would like to draw attention to this significant and preventable public health problem. In addition, under-ascertainment of cases is also expected in these outbreaks, because not all listeriosis cases are diagnosed and isolates from diagnosed listeriosis cases are not always sent to the laboratory for sequencing. Therefore, we assume that the number of cases is substantially higher.

Even though listeriosis cases are often associated with the consumption of contaminated RTE food products, such as cheese, meat and fish products, graved, and smoked fish are the RTE foods most frequently contaminated with *L. monocytogenes* around the world [21–24]. Salmon and salmon products are therefore seen as an important source of human exposure with *L. monocytogenes* in other countries as well [25,26].

In notifications of the Rapid Alert System for Food and Feed (RASFF) affecting Germany, fish was the second most frequently reported food product contaminated with *L. monocytogenes* after dairy products between 2001 and 2015 [27]. Appropriate food safety measures are necessary at all levels in order to minimize the risk of listeriosis from smoked or graved salmon products. Independent from the outbreaks in this report, experiences in Sweden for example, indicate that fish-processing plants need enhanced guidance for *L. monocytogenes* control from the supervising authorities, and continuous control measures and testing for *L. monocytogenes* at the plant should be implemented [28,29]. Also, in other European countries, isolates from cases of listeriosis have been identified that are closely related to many of the outbreaks described here. This is not unexpected as salmon products are often produced, processed, and sold internationally and listeriosis outbreaks linked to salmon products commonly affect several countries [9,17]. Therefore, comprehensive risk management is required not only in Germany but also internationally, in order to identify the persistence of *L. monocytogenes* in fish-processing plants, to stop the contamination of fish products and the distribution of Listeria across borders internationally operating fish-producing companies.

A minimum of eight out of the 22 outbreaks include cases in 2021 and therefore have to be assumed as ongoing. The persistent occurrence of cases is an indication for continuous contamination in production facilities and further listeriosis cases are to be expected.

In Germany, the incriminated products are mainly sold in superregional grocery chains. These should be made aware of the Listeria risks shown in our report. Special precautionary measures should be taken in the selection of supplies and quality assurance for these risk products.

Due to the higher listeriosis risk in smoked and graved salmon products, these should not be offered for consumption to vulnerable subpopulations, such as immunosuppressed patients and old people in healthcare facilities. In addition, dietary recommendations for the elderly correctly point out the positive health effects of fish but in these publications, the information on microbial risks of smoked and graved salmon is rarely mentioned. Due to the public health urgency, BfR and RKI published a German article about the outbreaks in the national bulletin [30].

Producers of smoked and graved salmon need to become more aware of their food safety responsibilities with regard to Listeria and intensify their efforts to minimize the entry, spread, and persistence of the pathogen in the production environment and thus the contamination of products. Smoked or graved salmon products contaminated with Listeria should not be placed on the market or should be withdrawn if they are already put on sale.

Beginning in 2018, WGS-based molecular surveillance of *L. monocytogenes* infections was established in Germany and since then all clinical Listeria isolates are analysed by WGS. This has proven highly valuable for outbreak detection and investigation as illustrated by both previous outbreak investigations and this report.

Developing a future system for easy exchange and comparison of WGS data across borders would allow for the identification of more dispersed outbreaks as well as cross-border linking of contaminated food samples and human infections [17]. Routine real-time data sharing would minimize human exposure to contaminated food products and save lives by reducing the time a foodborne pathogen is present in the global food chain [31]. Food production and trade practice call for an international collaborating network with a One-Health approach to facilitate global disease surveillance and early recognition and avoidance of foodborne outbreaks.

## Supplementary Material

Supplemental Material
