# Supplementary material for: Invasive listeriosis outbreaks and salmon products: a genomic, epidemiological study
Source: Emerg Microbes Infect. 2022 May 23;11(1):1308–15. doi: 10.1080/22221751.2022.2063075 (PMC9132468; doi:10.1080/22221751.2022.2063075)
Supplement: Supplemental Material [file TEMI_A_2063075_SM3604.docx]

Supplemental Table 1: **Characteristics of the isolates used in this study, including ENA accession numbers**

| **Isolate** | **Source Type** | **Cluster** | **Collection year** | **serogroup** | **MLST ST** | **clonal complex** | **cgMLST CT** | **Perc. Good Targets** | **Sample Accession** | **Study** |
| --- | --- | --- | --- | --- | --- | --- | --- | --- | --- | --- |
| 16-00861 | clinical/host-associated | Alpha4 | 2016 | IIa | 8 | CC8 | 1269 | 99.5 | SAMEA104485245 | PRJEB24496 |
| 17-05940 | clinical/host-associated | Alpha4 | 2017 | IIa | 8 | CC8 | 1269 | 92.9 | SAMEA6800875 | PRJEB37942 |
| 19-04821 | clinical/host-associated | Alpha4 | 2019 | IIa | 8 | CC8 | 1269 | 98.3 | SAMEA6602597 | PRJEB29295 |
| 19-05172 | clinical/host-associated | Alpha4 | 2019 | IIa | 8 | CC8 | 1269 | 100.0 | SAMEA6245406 | PRJEB29295 |
| 20-01417 | clinical/host-associated | Alpha4 | 2020 | IIa | 8 | CC8 | 1269 | 99.8 | SAMEA10018410 | PRJEB48063 |
| 16-LI00781-0 | Food | Alpha4 | 2016 | IIa | 8 | CC8 | 1269 | 100.0 | SAMEA6855929 | PRJEB38495 |
| 17-LI00548-0 | Food | Alpha4 | 2017 | IIa | 8 | CC8 | 1269 | 100.0 | SAMN22043160 | PRJNA768430 |
| 16-03732 | clinical/host-associated | Beta2a | 2016 | IIa | 8 | CC8 | 1247 | 99.8 | SAMEA104485369 | PRJEB24496 |
| 17-00451 | clinical/host-associated | Beta2a | 2017 | IIa | 8 | CC8 | 1247 | 99.8 | SAMEA104485457 | PRJEB24496 |
| 17-00642 | clinical/host-associated | Beta2a | 2017 | IIa | 8 | CC8 | 1247 | 99.8 | SAMEA10018378 | PRJEB48063 |
| 18-03379 | clinical/host-associated | Beta2a | 2018 | IIa | 8 | CC8 | 1247 | 98.4 | SAMEA6800953 | PRJEB37942 |
| 18-03580 | clinical/host-associated | Beta2a | 2018 | IIa | 8 | CC8 | 1247 | 100.0 | SAMEA10017316 | PRJEB48063 |
| 20-01419 | clinical/host-associated | Beta2a | 2020 | IIa | 8 | CC8 | 1247 | 100.0 | SAMEA10018411 | PRJEB48063 |
| 20-02467 | clinical/host-associated | Beta2a | 2020 | IIa | 8 | CC8 | 1247 | 99.6 | SAMEA10017381 | PRJEB48063 |
| 21-00973 | clinical/host-associated | Beta2a | 2021 | IIa | 8 | CC8 | 1247 | 99.8 | SAMEA10017404 | PRJEB48063 |
| 21-01554 | clinical/host-associated | Beta2a | 2021 | IIa | 8 | CC8 | 1247 | 99.7 | SAMEA10017407 | PRJEB48063 |
| 21-02068 | clinical/host-associated | Beta2a | 2021 | IIa | 8 | CC8 | 1247 | 95.0 | SAMEA10017410 | PRJEB48063 |
| 21-02143 | clinical/host-associated | Beta2a | 2021 | IIa | 8 | CC8 | 1247 | 99.8 | SAMEA10017411 | PRJEB48063 |
| 21-02343 | clinical/host-associated | Beta2a | 2021 | IIa | 8 | CC8 | 1247 | 99.1 | SAMEA10017413 | PRJEB48063 |
| 21-02715 | clinical/host-associated | Beta2a | 2021 | IIa | 8 | CC8 | 1247 | 99.2 | SAMEA10017415 | PRJEB48063 |
| 13-LI00405-0 | Food | Beta2a | 2013 | IIa | 8 | CC8 | 1247 | 99.9 | SAMN22043161 | PRJNA768430 |
| 17-LI00702-0 | Food | Beta2a | 2017 | IIa | 8 | CC8 | 1247 | 91.7 | SAMN22043162 | PRJNA768430 |
| 17-LI01057-0 | Food | Beta2a | 2017 | IIa | 8 | CC8 | 1247 | 99.9 | SAMN22043163 | PRJNA768430 |
| 18-LI00027-0 | Food | Beta2a | 2017 | IIa | 8 | CC8 | 1247 | 99.9 | SAMN22043164 | PRJNA768430 |
| 18-LI01156-0 | Food | Beta2a | 2018 | IIa | 8 | CC8 | 1247 | 99.9 | SAMN22043165 | PRJNA768430 |
| 2011-485 | Food | Beta2a | 2011 | IIa | 8 | CC8 | 1247 | 100.0 | SAMN22043166 | PRJNA768430 |
| 20-LI01284-0 | Food | Beta2a | 2020 | IIa | 8 | CC8 | 1247 | 99.9 | SAMN22043167 | PRJNA768430 |
| 21-LI00175-0 | Food | Beta2a | 2020 | IIa | 8 | CC8 | 1247 | 99.9 | SAMN22043168 | PRJNA768430 |
| 21-LI00190-0 | Food | Beta2a | 2021 | IIa | 8 | CC8 | 1247 | 99.9 | SAMN22043169 | PRJNA768430 |
| 21-LI00192-0 | Food | Beta2a | 2021 | IIa | 8 | CC8 | 1247 | 99.9 | SAMN22043170 | PRJNA768430 |
| 21-LI00193-0 | Food | Beta2a | 2021 | IIa | 8 | CC8 | 1247 | 99.9 | SAMN22043171 | PRJNA768430 |
| 21-LI00338-0 | Food | Beta2a | 2021 | IIa | 8 | CC8 | 1247 | 99.9 | SAMN22043172 | PRJNA768430 |
| 21-LI00401-0 | Food | Beta2a | 2021 | IIa | 8 | CC8 | 1247 | 99.9 | SAMN22043173 | PRJNA768430 |
| 21-LI00405-0 | Food | Beta2a | 2021 | IIa | 8 | CC8 | 1247 | 99.9 | SAMN22043174 | PRJNA768430 |
| 21-LI00410-0 | Food | Beta2a | 2021 | IIa | 8 | CC8 | 1247 | 99.9 | SAMN22043175 | PRJNA768430 |
| 16-00304 | clinical/host-associated | Chi1a | 2016 | IIa | 14 | CC14 | 2966 | 99.5 | SAMEA104485220 | PRJEB24496 |
| 16-01823 | clinical/host-associated | Chi1a | 2016 | IIa | 14 | CC14 | 2966 | 99.5 | SAMEA104485281 | PRJEB24496 |
| 16-02583 | clinical/host-associated | Chi1a | 2016 | IIa | 14 | CC14 | 2966 | 99.3 | SAMEA10018375 | PRJEB48063 |
| 16-02588 | clinical/host-associated | Chi1a | 2016 | IIa | 14 | CC14 | 2966 | 99.5 | SAMEA10018377 | PRJEB48063 |
| 16-02944 | clinical/host-associated | Chi1a | 2016 | IIa | 14 | CC14 | 2966 | 99.5 | SAMEA104485331 | PRJEB24496 |
| 17-02335 | clinical/host-associated | Chi1a | 2017 | IIa | 14 | CC14 | 5583 | 99.4 | SAMEA10017298 | PRJEB48063 |
| 18-00517 | clinical/host-associated | Chi1a | 2017 | IIa | 14 | CC14 | 2966 | 99.7 | SAMEA10018422 | PRJEB48063 |
| 18-03579 | clinical/host-associated | Chi1a | 2018 | IIa | 14 | CC14 | 2966 | 98.8 | SAMEA10017315 | PRJEB48063 |
| 18-04447 | clinical/host-associated | Chi1a | 2018 | IIa | 14 | CC14 | 2966 | 99.9 | SAMEA10017323 | PRJEB48063 |
| 19-05964 | clinical/host-associated | Chi1a | 2019 | IIa | 14 | CC14 | 2966 | 99.8 | SAMEA10017348 | PRJEB48063 |
| 19-06323 | clinical/host-associated | Chi1a | 2019 | IIa | 14 | CC14 | 2966 | 99.5 | SAMEA10017351 | PRJEB48063 |
| 20-00481 | clinical/host-associated | Chi1a | 2019 | IIa | 14 | CC14 | 2966 | 99.8 | SAMEA10018427 | PRJEB48063 |
| 14-LI00660-0 | Food | Chi1a | 2014 | IIa | 14 | CC14 | 2966 | 99.9 | SAMN22043176 | PRJNA768430 |
| 14-LI00762-0 | Food | Chi1a | 2014 | IIa | 14 | CC14 | 2966 | 99.8 | SAMN22043177 | PRJNA768430 |
| 15-LI00340-0 | Food | Chi1a | 2015 | IIa | 14 | CC14 | 2966 | 95.2 | SAMN22043178 | PRJNA768430 |
| 16-LI00804-0 | Food | Chi1a | 2016 | IIa | 14 | CC14 | 2966 | 99.9 | SAMEA6855944 | PRJEB38495 |
| 16-LI00808-0 | Food | Chi1a | 2016 | IIa | 14 | CC14 | 2966 | 99.9 | SAMEA6855945 | PRJEB38495 |
| 16-LI01075-1 | Food | Chi1a | 2016 | IIa | 14 | CC14 | 2966 | 99.9 | SAMEA6856103 | PRJEB38495 |
| 17-LI00706-0 | Food | Chi1a | 2017 | IIa | 14 | CC14 | 2966 | 99.9 | SAMN22043179 | PRJNA768430 |
| 17-LI01041-0 | Food | Chi1a | 2017 | IIa | 14 | CC14 | 2966 | 99.8 | SAMN22043180 | PRJNA768430 |
| 2012-387 | Food | Chi1a | 2012 | IIa | 14 | CC14 | 2966 | 99.9 | SAMN22043181 | PRJNA768430 |
| 18-00477 | clinical/host-associated | Chi4 | 2018 | IIa | 451 | CC451 | 4035 | 99.6 | SAMEA6602598 | PRJEB29295 |
| 18-01856 | clinical/host-associated | Chi4 | 2018 | IIa | 451 | CC451 | 4035 | 99.1 | SAMEA10018399 | PRJEB48063 |
| 16-LI01095-0 | Food | Chi4 | 2016 | IIa | 451 | CC451 | 4035 | 99.9 | SAMEA6856114 | PRJEB38495 |
| 19-06683 | clinical/host-associated | Chi6b | 2019 | IVb | 6 | CC6 | 1738 | 98.1 | SAMEA10017356 | PRJEB48063 |
| 19-06992 | clinical/host-associated | Chi6b | 2019 | IVb | 6 | CC6 | 1738 | 99.6 | SAMEA10017359 | PRJEB48063 |
| 19-07017 | clinical/host-associated | Chi6b | 2019 | IVb | 6 | CC6 | 1738 | 99.2 | SAMEA10017360 | PRJEB48063 |
| 19-07167 | clinical/host-associated | Chi6b | 2019 | IVb | 6 | CC6 | 9071 | 97.1 | SAMEA10017363 | PRJEB48063 |
| 19-07325 | clinical/host-associated | Chi6b | 2019 | IVb | 6 | CC6 | 1738 | 99.6 | SAMEA7540779 | PRJEB29295 |
| 16-LI00751-0 | Food | Chi6b | 2016 | IVb | 6 | CC6 | 1738 | 99.6 | SAMEA6855918 | PRJEB38495 |
| 13-03594 | clinical/host-associated | Delta1 | 2013 | IVb | 6 | CC6 | 3530 | 99.1 | SAMEA104485080 | PRJEB24496 |
| 15-03034 | clinical/host-associated | Delta1 | 2015 | IVb | 6 | CC6 | 3530 | 99.2 | SAMEA104485187 | PRJEB24496 |
| 15-04491 | clinical/host-associated | Delta1 | 2015 | IVb | 6 | CC6 | 3530 | 99.1 | SAMEA104485200 | PRJEB24496 |
| 16-01191 | clinical/host-associated | Delta1 | 2016 | IVb | 6 | CC6 | 3530 | 99.2 | SAMEA104485252 | PRJEB24496 |
| 16-01236 | clinical/host-associated | Delta1 | 2016 | IVb | 6 | CC6 | 3530 | 99.3 | SAMEA104485255 | PRJEB24496 |
| 16-01400 | clinical/host-associated | Delta1 | 2016 | IVb | 6 | CC6 | 3530 | 99.1 | SAMEA104485261 | PRJEB24496 |
| 16-01418 | clinical/host-associated | Delta1 | 2016 | IVb | 6 | CC6 | 3530 | 99.2 | SAMEA104485264 | PRJEB24496 |
| 16-01677 | clinical/host-associated | Delta1 | 2016 | IVb | 6 | CC6 | 3530 | 99.1 | SAMEA104485278 | PRJEB24496 |
| 16-02006 | clinical/host-associated | Delta1 | 2016 | IVb | 6 | CC6 | 3530 | 99.2 | SAMEA104485288 | PRJEB24496 |
| 16-02187 | clinical/host-associated | Delta1 | 2016 | IVb | 6 | CC6 | 3530 | 99.1 | SAMEA104485294 | PRJEB24496 |
| 16-02327 | clinical/host-associated | Delta1 | 2015 | IVb | 6 | CC6 | 3530 | 99.2 | SAMEA104485300 | PRJEB24496 |
| 16-04004 | clinical/host-associated | Delta1 | 2016 | IVb | 6 | CC6 | 3530 | 99.3 | SAMEA104485388 | PRJEB24496 |
| 16-04332 | clinical/host-associated | Delta1 | 2016 | IVb | 6 | CC6 | 3530 | 99.2 | SAMEA104485405 | PRJEB24496 |
| 16-04493 | clinical/host-associated | Delta1 | 2016 | IVb | 6 | CC6 | 3530 | 99.3 | SAMEA104485412 | PRJEB24496 |
| 16-04680 | clinical/host-associated | Delta1 | 2016 | IVb | 6 | CC6 | 3530 | 99.1 | SAMEA104485418 | PRJEB24496 |
| 16-04923 | clinical/host-associated | Delta1 | 2016 | IVb | 6 | CC6 | 3530 | 99.2 | SAMEA104485426 | PRJEB24496 |
| 17-00223 | clinical/host-associated | Delta1 | 2017 | IVb | 6 | CC6 | 3530 | 99.1 | SAMEA104485445 | PRJEB24496 |
| 17-00380 | clinical/host-associated | Delta1 | 2017 | IVb | 6 | CC6 | 3530 | 99.2 | SAMEA104485452 | PRJEB24496 |
| 17-00400 | clinical/host-associated | Delta1 | 2017 | IVb | 6 | CC6 | 3530 | 99.1 | SAMEA104485453 | PRJEB24496 |
| 17-00673 | clinical/host-associated | Delta1 | 2017 | IVb | 6 | CC6 | 3530 | 99.2 | SAMEA10018379 | PRJEB48063 |
| 17-02334 | clinical/host-associated | Delta1 | 2017 | IVb | 6 | CC6 | 3530 | 99.1 | SAMEA10018387 | PRJEB48063 |
| 18-07098 | clinical/host-associated | Delta1 | 2018 | IVb | 6 | CC6 | 3530 | 99.6 | SAMEA10018403 | PRJEB48063 |
| 13-LI00247-0 | Food | Delta1 | 2013 | IVb | 6 | CC6 | 3530 | 99.5 | SAMN22043182 | PRJNA768430 |
| 16-LI00426-0 | Food | Delta1 | 2016 | IVb | 6 | CC6 | 3530 | 99.6 | SAMEA6855819 | PRJEB38495 |
| 16-LI00427-0 | Food | Delta1 | 2016 | IVb | 6 | CC6 | 3530 | 99.6 | SAMEA6855820 | PRJEB38495 |
| 16-LI01104-0 | Food | Delta1 | 2016 | IVb | 6 | CC6 | 3530 | 99.6 | SAMEA6856120 | PRJEB38495 |
| 18-LI00836-0 | Food | Delta1 | 2018 | IVb | 6 | CC6 | 3530 | 98.4 | SAMN22043183 | PRJNA768430 |
| 18-05656 | clinical/host-associated | Delta8 | 2018 | IIa | 121 | CC121 | 4295 | 99.8 | SAMEA6800726 | PRJEB37942 |
| 19-06774 | clinical/host-associated | Delta8 | 2019 | IIa | 121 | CC121 | 4295 | 99.9 | SAMEA10017358 | PRJEB48063 |
| 20-00385 | clinical/host-associated | Delta8 | 2020 | IIa | 121 | CC121 | 4295 | 99.6 | SAMEA10017370 | PRJEB48063 |
| 20-02684 | clinical/host-associated | Delta8 | 2020 | IIa | 121 | CC121 | 4295 | 99.6 | SAMEA7540790 | PRJEB29295 |
| 13-LI00591-0 | Food | Delta8 | 2013 | IIa | 121 | CC121 | 4295 | 99.9 | SAMN22043184 | PRJNA768430 |
| 17-LI00910-0 | Food | Delta8 | 2017 | IIa | 121 | CC121 | 4295 | 99.9 | SAMN22043185 | PRJNA768430 |
| 16-03100 | clinical/host-associated | Eta5 | 2016 | IIa | 37 | CC37 | 5488 | 99.8 | SAMEA10017297 | PRJEB48063 |
| 17-03525 | clinical/host-associated | Eta5 | 2017 | IIa | 37 | CC37 | 5488 | 99.8 | SAMEA6800616 | PRJEB37942 |
| 19-03437 | clinical/host-associated | Eta5 | 2019 | IIa | ? | ? | 5488 | 99.8 | SAMEA10017340 | PRJEB48063 |
| 19-03903 | clinical/host-associated | Eta5 | 2019 | IIa | 37 | CC37 | 5488 | 99.3 | SAMEA6602609 | PRJEB29295 |
| 19-04324 | clinical/host-associated | Eta5 | 2019 | IIa | 37 | CC37 | 5488 | 99.5 | SAMEA10017343 | PRJEB48063 |
| 20-00157 | clinical/host-associated | Eta5 | 2020 | IIa | 37 | CC37 | 5488 | 99.9 | SAMEA10017366 | PRJEB48063 |
| 20-00163-1 | clinical/host-associated | Eta5 | 2020 | IIa | 37 | CC37 | 5488 | 99.9 | SAMEA10017367 | PRJEB48063 |
| 16-LI00820-0 | Food | Eta5 | 2016 | IIa | 37 | CC37 | 5488 | 98.8 | SAMEA6855951 | PRJEB38495 |
| 17-LI00504-0 | Food | Eta5 | 2017 | IIa | 37 | CC37 | 5488 | 99.9 | SAMN22043186 | PRJNA768430 |
| 17-07026 | clinical/host-associated | Eta8 | 2017 | IIa | 37 | CC37 | 4230 | 99.9 | SAMEA6800904 | PRJEB37942 |
| 19-07147 | clinical/host-associated | Eta8 | 2019 | IIa | 37 | CC37 | 4230 | 99.1 | SAMEA10017362 | PRJEB48063 |
| 20-06277 | clinical/host-associated | Eta8 | 2020 | IIa | 37 | CC37 | 4230 | 99.8 | SAMEA10018420 | PRJEB48063 |
| 21-00134 | clinical/host-associated | Eta8 | 2021 | IIa | 37 | CC37 | 4230 | 99.8 | SAMEA10017401 | PRJEB48063 |
| 16-LI00783-0 | Food | Eta8 | 2016 | IIa | 37 | CC37 | 4230 | 99.9 | SAMEA6855931 | PRJEB38495 |
| 16-LI01066-0 | Food | Eta8 | 2016 | IIa | 37 | CC37 | 4230 | 99.9 | SAMEA6856094 | PRJEB38495 |
| 17-05202 | clinical/host-associated | Iota1c | 2017 | IIa | 8 | CC8 | 73 | 99.6 | SAMEA6800858 | PRJEB37942 |
| 17-05929 | clinical/host-associated | Iota1c | 2017 | IIa | 8 | CC8 | 73 | 94.7 | SAMEA10018394 | PRJEB48063 |
| 18-03448 | clinical/host-associated | Iota1c | 2018 | IIa | 8 | CC8 | 6756 | 100.0 | SAMEA10017314 | PRJEB48063 |
| 20-00435 | clinical/host-associated | Iota1c | 2020 | IIa | 8 | CC8 | 73 | 100.0 | SAMEA7540791 | PRJEB29295 |
| 20-00464 | clinical/host-associated | Iota1c | 2019 | IIa | 8 | CC8 | 73 | 99.9 | SAMEA10018426 | PRJEB48063 |
| 16-LI00843-0 | Food | Iota1c | 2016 | IIa | 8 | CC8 | 1366 | 100.0 | SAMEA6855961 | PRJEB38495 |
| 17-00157 | clinical/host-associated | My2 | 2017 | IIa | 173 | CC19 | 3242 | 99.6 | SAMEA104485443 | PRJEB24496 |
| 17-00169 | clinical/host-associated | My2 | 2017 | IIa | 173 | CC19 | 3242 | 99.5 | SAMEA104485444 | PRJEB24496 |
| 17-01914 | clinical/host-associated | My2 | 2017 | IIa | 173 | CC19 | 3242 | 99.6 | SAMEA10018384 | PRJEB48063 |
| 17-02550 | clinical/host-associated | My2 | 2017 | IIa | 173 | CC19 | 3242 | 99.5 | SAMEA10017300 | PRJEB48063 |
| 17-05750 | clinical/host-associated | My2 | 2017 | IIa | 173 | CC19 | 3242 | 99.4 | SAMEA10018391 | PRJEB48063 |
| 17-06729 | clinical/host-associated | My2 | 2017 | IIa | 173 | CC19 | 3242 | 99.7 | SAMEA6602599 | PRJEB29295 |
| 19-00081 | clinical/host-associated | My2 | 2019 | IIa | 173 | CC19 | 3242 | 99.8 | SAMEA10017331 | PRJEB48063 |
| 19-00663 | clinical/host-associated | My2 | 2019 | IIa | 173 | CC19 | 3242 | 99.6 | SAMEA10018406 | PRJEB48063 |
| 19-01821 | clinical/host-associated | My2 | 2019 | IIa | 173 | CC19 | 3242 | 99.8 | SAMEA10017337 | PRJEB48063 |
| 19-01986 | clinical/host-associated | My2 | 2019 | IIa | 173 | CC19 | 3242 | 92.5 | SAMEA10017338 | PRJEB48063 |
| 19-05170 | clinical/host-associated | My2 | 2019 | IIa | 173 | CC19 | 3242 | 99.8 | SAMEA10017344 | PRJEB48063 |
| 19-05967 | clinical/host-associated | My2 | 2019 | IIa | 173 | CC19 | 3242 | 93.4 | SAMEA10017349 | PRJEB48063 |
| 19-06322 | clinical/host-associated | My2 | 2019 | IIa | 173 | CC19 | 3242 | 99.6 | SAMEA10017350 | PRJEB48063 |
| 19-06389 | clinical/host-associated | My2 | 2019 | IIa | 173 | CC19 | 3242 | 99.3 | SAMEA10017352 | PRJEB48063 |
| 20-01090 | clinical/host-associated | My2 | 2020 | IIa | 173 | CC19 | 3242 | 99.2 | SAMEA10017376 | PRJEB48063 |
| 20-01163 | clinical/host-associated | My2 | 2020 | IIa | 173 | CC19 | 3242 | 98.9 | SAMEA10017377 | PRJEB48063 |
| 20-01851 | clinical/host-associated | My2 | 2020 | IIa | 173 | CC19 | 3242 | 99.6 | SAMEA10018414 | PRJEB48063 |
| 20-06420 | clinical/host-associated | My2 | 2020 | IIa | 173 | CC19 | 3242 | 99.2 | SAMEA10017398 | PRJEB48063 |
| 21-01649 | clinical/host-associated | My2 | 2021 | IIa | 173 | CC19 | 3242 | 99.7 | SAMEA10017408 | PRJEB48063 |
| 16-LI00385-0 | Food | My2 | 2016 | IIa | 173 | CC19 | 3242 | 99.8 | SAMEA6855800 | PRJEB38495 |
| 18-LI00056-0 | Food | My2 | 2017 | IIa | 173 | CC19 | 3242 | 99.6 | SAMN22043187 | PRJNA768430 |
| 21-LI00065-0 | Food | My2 | 2020 | IIa | 173 | CC19 | 3242 | 99.7 | SAMN22043190 | PRJNA768430 |
| 21-LI00383-0 | Food | My2 | 2021 | IIa | 173 | CC19 | 3242 | 99.7 | SAMN22043191 | PRJNA768430 |
| 21-LI00549-0 | Food | My2 | 2021 | IIa | 173 | CC19 | 3242 | 99.7 | SAMN22043193 | PRJNA768430 |
| 21-LI00572-0 | Food | My2 | 2021 | IIa | 173 | CC19 | 3242 | 99.6 | SAMN22043198 | PRJNA768430 |
| 20-LI01174-0 | Food processing environment | My2 | 2020 | IIa | 173 | CC19 | 3242 | 99.7 | SAMN22043188 | PRJNA768430 |
| 20-LI01269-0 | Food processing environment | My2 | 2020 | IIa | 173 | CC19 | 3242 | 99.7 | SAMN22043189 | PRJNA768430 |
| 21-LI00530-1 | Food processing environment | My2 | 2021 | IIa | 173 | CC19 | 3242 | 99.6 | SAMN22043192 | PRJNA768430 |
| 21-LI00568-0 | Food processing environment | My2 | 2021 | IIa | 173 | CC19 | 3242 | 99.7 | SAMN22043194 | PRJNA768430 |
| 21-LI00569-0 | Food processing environment | My2 | 2021 | IIa | 173 | CC19 | 3242 | 99.6 | SAMN22043195 | PRJNA768430 |
| 21-LI00570-0 | Food processing environment | My2 | 2021 | IIa | 173 | CC19 | 3242 | 99.7 | SAMN22043196 | PRJNA768430 |
| 21-LI00571-0 | Food processing environment | My2 | 2021 | IIa | 173 | CC19 | 3242 | 99.7 | SAMN22043197 | PRJNA768430 |
| 21-LI00727-0 | Food processing environment | My2 | 2021 | IIa | 173 | CC19 | 3242 | 99.7 | SAMN22043199 | PRJNA768430 |
| 21-LI00728-0 | Food processing environment | My2 | 2021 | IIa | 173 | CC19 | 3242 | 99.7 | SAMN22043200 | PRJNA768430 |
| 21-LI00729-0 | Food processing environment | My2 | 2021 | IIa | 173 | CC19 | 3242 | 99.7 | SAMN22043201 | PRJNA768430 |
| 21-LI00730-0 | Food processing environment | My2 | 2021 | IIa | 173 | CC19 | 3242 | 99.7 | SAMN22043202 | PRJNA768430 |
| 21-LI00731-0 | Food processing environment | My2 | 2021 | IIa | 173 | CC19 | 3242 | 99.6 | SAMN22043203 | PRJNA768430 |
| 21-LI00732-0 | Food processing environment | My2 | 2021 | IIa | 173 | CC19 | 3242 | 99.7 | SAMN22043204 | PRJNA768430 |
| 18-04854 | clinical/host-associated | Omega5 | 2018 | IIb | 87 | CC87 | 773 | 99.4 | SAMEA6602600 | PRJEB29295 |
| 18-05039 | clinical/host-associated | Omega5 | 2018 | IIb | 87 | CC87 | 1138 | 98.7 | SAMEA10017325 | PRJEB48063 |
| 19-03440 | clinical/host-associated | Omega5 | 2019 | IIb | 87 | CC87 | 1138 | 99.4 | SAMEA10017341 | PRJEB48063 |
| 19-03740 | clinical/host-associated | Omega5 | 2019 | IIb | 87 | CC87 | 1138 | 99.4 | SAMEA10017342 | PRJEB48063 |
| 19-05237 | clinical/host-associated | Omega5 | 2019 | IIb | 2891 | CC87 | 1138 | 99.4 | SAMEA10017345 | PRJEB48063 |
| 19-05347 | clinical/host-associated | Omega5 | 2019 | IIb | 87 | CC87 | 1138 | 99.4 | SAMEA10017346 | PRJEB48063 |
| 19-05348 | clinical/host-associated | Omega5 | 2019 | IIb | 87 | CC87 | 1138 | 99.1 | SAMEA10017347 | PRJEB48063 |
| 20-02625 | clinical/host-associated | Omega5 | 2020 | IIb | 87 | CC87 | 1138 | 98.7 | SAMEA10017383 | PRJEB48063 |
| 20-03178 | clinical/host-associated | Omega5 | 2020 | IIb | 87 | CC87 | 773 | 97.5 | SAMEA10017388 | PRJEB48063 |
| 20-06276 | clinical/host-associated | Omega5 | 2020 | IIb | 87 | CC87 | 1138 | 98.8 | SAMEA10017396 | PRJEB48063 |
| 20-06341 | clinical/host-associated | Omega5 | 2020 | IIb | 87 | CC87 | 1138 | 98.8 | SAMEA10017397 | PRJEB48063 |
| 16-LI00959-0 | Food | Omega5 | 2016 | IIb | 87 | CC87 | 535 | 99.4 | SAMEA6856035 | PRJEB38495 |
| 16-LI01081-0 | Food | Omega5 | 2016 | IIb | 87 | CC87 | 535 | 99.4 | SAMEA6856109 | PRJEB38495 |
| 18-LI01101-0 | Food | Omega5 | 2018 | IIb | 87 | CC87 | 1138 | 99.4 | SAMN22043205 | PRJNA768430 |
| 21-LI00155-0 | Food | Omega5 | 2020 | IIb | 87 | CC87 | 1138 | 98.8 | SAMN22043206 | PRJNA768430 |
| 21-LI00474-0 | Food | Omega5 | 2021 | IIb | 87 | CC87 | 1138 | 98.8 | SAMN22043207 | PRJNA768430 |
| 16-00494 | clinical/host-associated | Omikron1 | 2016 | IIa | 155 | CC155 | 1128 | 99.5 | SAMEA104485233 | PRJEB24496 |
| 16-02584 | clinical/host-associated | Omikron1 | 2016 | IIa | 155 | CC155 | 1128 | 99.5 | SAMEA10018376 | PRJEB48063 |
| 16-03805 | clinical/host-associated | Omikron1 | 2016 | IIa | 155 | CC155 | 1128 | 99.5 | SAMEA104485375 | PRJEB24496 |
| 16-05275 | clinical/host-associated | Omikron1 | 2016 | IIa | 155 | CC155 | 1128 | 99.5 | SAMEA104485434 | PRJEB24496 |
| 17-00305 | clinical/host-associated | Omikron1 | 2017 | IIa | 155 | CC155 | 1128 | 99.5 | SAMEA104485449 | PRJEB24496 |
| 17-01049 | clinical/host-associated | Omikron1 | 2017 | IIa | 155 | CC155 | 1128 | 99.5 | SAMEA6800785 | PRJEB37942 |
| 17-01288 | clinical/host-associated | Omikron1 | 2017 | IIa | 155 | CC155 | 1128 | 99.6 | SAMEA10018380 | PRJEB48063 |
| 17-01316 | clinical/host-associated | Omikron1 | 2017 | IIa | 155 | CC155 | 1128 | 99.5 | SAMEA10018381 | PRJEB48063 |
| 17-01589 | clinical/host-associated | Omikron1 | 2017 | IIa | 155 | CC155 | 1128 | 99.5 | SAMEA10018383 | PRJEB48063 |
| 17-02120 | clinical/host-associated | Omikron1 | 2017 | IIa | 155 | CC155 | 1128 | 99.6 | SAMEA10018385 | PRJEB48063 |
| 17-02487 | clinical/host-associated | Omikron1 | 2017 | IIa | 155 | CC155 | 1128 | 99.5 | SAMEA10017299 | PRJEB48063 |
| 17-03598 | clinical/host-associated | Omikron1 | 2017 | IIa | 155 | CC155 | 1128 | 99.7 | SAMEA10017302 | PRJEB48063 |
| 17-04407 | clinical/host-associated | Omikron1 | 2017 | IIa | 155 | CC155 | 1128 | 99.6 | SAMEA10017305 | PRJEB48063 |
| 17-04736 | clinical/host-associated | Omikron1 | 2017 | IIa | 155 | CC155 | 1128 | 99.6 | SAMEA10017306 | PRJEB48063 |
| 17-04778 | clinical/host-associated | Omikron1 | 2017 | IIa | 155 | CC155 | 1128 | 99.7 | SAMEA10017307 | PRJEB48063 |
| 17-05075 | clinical/host-associated | Omikron1 | 2017 | IIa | 155 | CC155 | 1128 | 99.7 | SAMEA10017308 | PRJEB48063 |
| 17-05710 | clinical/host-associated | Omikron1 | 2017 | IIa | 155 | CC155 | 1128 | 91.6 | SAMEA10018390 | PRJEB48063 |
| 17-05866 | clinical/host-associated | Omikron1 | 2017 | IIa | 155 | CC155 | 1128 | 97.5 | SAMEA10018393 | PRJEB48063 |
| 18-04993 | clinical/host-associated | Omikron1 | 2018 | IIa | 155 | CC155 | 1128 | 99.5 | SAMEA10017324 | PRJEB48063 |
| 19-00901 | clinical/host-associated | Omikron1 | 2019 | IIa | ? | ? | 1128 | 91.2 | SAMEA10018407 | PRJEB48063 |
| 19-02320 | clinical/host-associated | Omikron1 | 2019 | IIa | 155 | CC155 | 1128 | 95.4 | SAMEA10017339 | PRJEB48063 |
| 19-06598 | clinical/host-associated | Omikron1 | 2019 | IIa | 155 | CC155 | 1128 | 99.4 | SAMEA10017354 | PRJEB48063 |
| 19-07744 | clinical/host-associated | Omikron1 | 2019 | IIa | 155 | CC155 | 1128 | 99.5 | SAMEA10017364 | PRJEB48063 |
| 20-00328 | clinical/host-associated | Omikron1 | 2020 | IIa | 155 | CC155 | 1128 | 99.5 | SAMEA10017369 | PRJEB48063 |
| 20-00558 | clinical/host-associated | Omikron1 | 2020 | IIa | 155 | CC155 | 1128 | 99.7 | SAMEA10017371 | PRJEB48063 |
| 20-00589 | clinical/host-associated | Omikron1 | 2020 | IIa | 155 | CC155 | 1128 | 99.6 | SAMEA10017372 | PRJEB48063 |
| 20-01269 | clinical/host-associated | Omikron1 | 2020 | IIa | 155 | CC155 | 1128 | 99.7 | SAMEA10017378 | PRJEB48063 |
| 20-01481 | clinical/host-associated | Omikron1 | 2020 | IIa | 155 | CC155 | 1128 | 99.6 | SAMEA10018412 | PRJEB48063 |
| 20-01786 | clinical/host-associated | Omikron1 | 2020 | IIa | 155 | CC155 | 1128 | 99.5 | SAMEA10018413 | PRJEB48063 |
| 20-01947 | clinical/host-associated | Omikron1 | 2020 | IIa | 155 | CC155 | 1128 | 99.5 | SAMEA10018415 | PRJEB48063 |
| 20-02178 | clinical/host-associated | Omikron1 | 2020 | IIa | 155 | CC155 | 1128 | 99.5 | SAMEA10017379 | PRJEB48063 |
| 20-02215 | clinical/host-associated | Omikron1 | 2020 | IIa | 155 | CC155 | 1128 | 97.9 | SAMEA10017380 | PRJEB48063 |
| 20-02496 | clinical/host-associated | Omikron1 | 2020 | IIa | 155 | CC155 | 1128 | 99.5 | SAMEA10017382 | PRJEB48063 |
| 20-02626 | clinical/host-associated | Omikron1 | 2020 | IIa | 155 | CC155 | 1128 | 99.4 | SAMEA10017384 | PRJEB48063 |
| 20-02627 | clinical/host-associated | Omikron1 | 2020 | IIa | 155 | CC155 | 1128 | 99.5 | SAMEA10017385 | PRJEB48063 |
| 20-02885 | clinical/host-associated | Omikron1 | 2020 | IIa | 155 | CC155 | 1128 | 99.5 | SAMEA10018416 | PRJEB48063 |
| 20-03720 | clinical/host-associated | Omikron1 | 2020 | IIa | 155 | CC155 | 1128 | 99.5 | SAMEA10017390 | PRJEB48063 |
| 20-05026 | clinical/host-associated | Omikron1 | 2020 | IIa | 155 | CC155 | 1128 | 99.6 | SAMEA10017395 | PRJEB48063 |
| 20-06303 | clinical/host-associated | Omikron1 | 2020 | IIa | 155 | CC155 | 1128 | 99.5 | SAMEA10018421 | PRJEB48063 |
| 21-00135 | clinical/host-associated | Omikron1 | 2021 | IIa | 155 | CC155 | 1128 | 99.5 | SAMEA10017402 | PRJEB48063 |
| 21-01318 | clinical/host-associated | Omikron1 | 2021 | IIa | 155 | CC155 | 1128 | 99.5 | SAMEA10017405 | PRJEB48063 |
| 21-01420 | clinical/host-associated | Omikron1 | 2021 | IIa | 155 | CC155 | 1128 | 99.5 | SAMEA10017406 | PRJEB48063 |
| 16-LI00911-0 | Food | Omikron1 | 2016 | IIa | 155 | CC155 | 1128 | 99.7 | SAMEA6856005 | PRJEB38495 |
| 16-LI01099-0 | Food | Omikron1 | 2016 | IIa | 155 | CC155 | 1128 | 99.7 | SAMEA6856117 | PRJEB38495 |
| 16-LI01162-0 | Food | Omikron1 | 2016 | IIa | 155 | CC155 | 1128 | 99.6 | SAMEA6856149 | PRJEB38495 |
| 17-LI00206-0 | Food | Omikron1 | 2017 | IIa | 155 | CC155 | 1128 | 99.7 | SAMN22043208 | PRJNA768430 |
| 17-LI00403-0 | Food | Omikron1 | 2017 | IIa | 155 | CC155 | 1128 | 99.5 | SAMN22043209 | PRJNA768430 |
| 17-LI00562-0 | Food | Omikron1 | 2017 | IIa | 155 | CC155 | 1128 | 99.6 | SAMN22043210 | PRJNA768430 |
| 17-LI00743-0 | Food | Omikron1 | 2017 | IIa | 155 | CC155 | 1128 | 99.6 | SAMN22043211 | PRJNA768430 |
| 20-LI00702-0 | Food | Omikron1 | 2020 | IIa | 155 | CC155 | 1128 | 99.5 | SAMN22043212 | PRJNA768430 |
| 20-LI00715-0 | Food | Omikron1 | 2020 | IIa | 155 | CC155 | 1128 | 99.5 | SAMN22043213 | PRJNA768430 |
| 20-LI00750-0 | Food | Omikron1 | 2020 | IIa | 155 | CC155 | 1128 | 99.5 | SAMN22043214 | PRJNA768430 |
| 20-LI01135-0 | Food | Omikron1 | 2020 | IIa | 155 | CC155 | 1128 | 99.5 | SAMN22043215 | PRJNA768430 |
| 21-LI00893-0 | Food | Omikron1 | 2021 | IIa | 155 | CC155 | 1128 | 99.5 | SAMN22043216 | PRJNA768430 |
| 17-03974 | clinical/host-associated | Omikron3 | 2017 | IIa | 8 | CC8 | 2994 | 100.0 | SAMEA10017303 | PRJEB48063 |
| 17-04065 | clinical/host-associated | Omikron3 | 2017 | IIa | 8 | CC8 | 2994 | 100.0 | SAMEA6602601 | PRJEB29295 |
| 17-04221 | clinical/host-associated | Omikron3 | 2017 | IIa | 8 | CC8 | 2994 | 100.0 | SAMEA10017304 | PRJEB48063 |
| 17-04323 | clinical/host-associated | Omikron3 | 2017 | IIa | 8 | CC8 | 2994 | 97.9 | SAMEA10018388 | PRJEB48063 |
| 17-07212 | clinical/host-associated | Omikron3 | 2017 | IIa | 8 | CC8 | 2994 | 99.5 | SAMEA10018396 | PRJEB48063 |
| 18-02511 | clinical/host-associated | Omikron3 | 2018 | IIa | 8 | CC8 | 4997 | 100.0 | SAMEA10017309 | PRJEB48063 |
| 18-05834 | clinical/host-associated | Omikron3 | 2018 | IIa | 8 | CC8 | 4997 | 99.9 | SAMEA10017328 | PRJEB48063 |
| 18-06544 | clinical/host-associated | Omikron3 | 2018 | IIa | 8 | CC8 | 4997 | 99.4 | SAMEA10017330 | PRJEB48063 |
| 09-04938 | clinical/host-associated | Rho3 | 2009 | IIc | 9 | CC9 | 1690 | 99.8 | SAMN03168662 | PRJNA266621 |
| 09-08048 | clinical/host-associated | Rho3 | 2009 | IIc | 9 | CC9 | 1690 | 99.8 | SAMN03168663 | PRJNA266621 |
| 10-01152 | clinical/host-associated | Rho3 | 2010 | IIc | 9 | CC9 | 1690 | 99.9 | SAMN03168659 | PRJNA266621 |
| 10-01814 | clinical/host-associated | Rho3 | 2010 | IIc | 9 | CC9 | 1690 | 99.9 | SAMN03168660 | PRJNA266621 |
| 11-00514 | clinical/host-associated | Rho3 | 2011 | IIc | 9 | CC9 | 1690 | 99.9 | SAMN03168658 | PRJNA266621 |
| 14-02475 | clinical/host-associated | Rho3 | 2014 | IIc | 9 | CC9 | 1690 | 99.9 | SAMN03168676 | PRJNA266621 |
| 17-01398 | clinical/host-associated | Rho3 | 2017 | IIc | 9 | CC9 | 1690 | 99.9 | SAMEA10018382 | PRJEB48063 |
| 18-00258 | clinical/host-associated | Rho3 | 2018 | IIa | 9 | CC9 | 1690 | 99.8 | SAMEA4920508 | PRJEB28663 |
| 18-00654 | clinical/host-associated | Rho3 | 2018 | IIc | 9 | CC9 | 1690 | 99.3 | SAMEA6602602 | PRJEB29295 |
| 18-03378 | clinical/host-associated | Rho3 | 2018 | IIc | 9 | CC9 | 1690 | 98.4 | SAMEA10018423 | PRJEB48063 |
| 18-03917 | clinical/host-associated | Rho3 | 2018 | IIc | 9 | CC9 | 1690 | 99.9 | SAMEA10017318 | PRJEB48063 |
| 14-LI00399-0 | Food | Rho3 | 2014 | IIa | 9 | CC9 | 1690 | 99.2 | SAMN22043217 | PRJNA768430 |
| 14-LI00694-0 | Food | Rho3 | 2014 | IIa | 9 | CC9 | 1690 | 99.4 | SAMN22043218 | PRJNA768430 |
| 16-LI00264-0 | Food | Rho3 | 2016 | IIc | 9 | CC9 | 1690 | 99.9 | SAMEA6855730 | PRJEB38495 |
| 16-LI00339-0 | Food | Rho3 | 2016 | IIc | 9 | CC9 | 1690 | 99.9 | SAMEA6855773 | PRJEB38495 |
| 16-LI00785-0 | Food | Rho3 | 2016 | IIc | 9 | CC9 | 1690 | 99.9 | SAMEA6855933 | PRJEB38495 |
| 16-LI00810-0 | Food | Rho3 | 2016 | IIc | 9 | CC9 | 1690 | 99.9 | SAMEA6855947 | PRJEB38495 |
| 16-LI00821-0 | Food | Rho3 | 2016 | IIc | 9 | CC9 | 1690 | 99.9 | SAMEA6855952 | PRJEB38495 |
| 16-LI00861-0 | Food | Rho3 | 2016 | IIc | 9 | CC9 | 1690 | 99.5 | SAMEA6855973 | PRJEB38495 |
| 16-LI00863-0 | Food | Rho3 | 2016 | IIc | 9 | CC9 | 1690 | 99.5 | SAMEA6855975 | PRJEB38495 |
| 16-LI00910-0 | Food | Rho3 | 2016 | IIc | 9 | CC9 | 1690 | 96.9 | SAMEA6856004 | PRJEB38495 |
| 16-LI00912-0 | Food | Rho3 | 2016 | IIa | 9 | CC9 | 1690 | 99.8 | SAMEA6856006 | PRJEB38495 |
| 16-LI00916-0 | Food | Rho3 | 2016 | IIc | 9 | CC9 | 1690 | 99.9 | SAMEA6856009 | PRJEB38495 |
| 16-LI00918-0 | Food | Rho3 | 2016 | IIc | 9 | CC9 | 1690 | 99.9 | SAMEA6856011 | PRJEB38495 |
| 16-LI00919-0 | Food | Rho3 | 2016 | IIc | 9 | CC9 | 1690 | 99.9 | SAMEA6856013 | PRJEB38495 |
| 16-LI00920-0 | Food | Rho3 | 2016 | IIc | 9 | CC9 | 1690 | 99.9 | SAMEA6856014 | PRJEB38495 |
| 16-LI00960-0 | Food | Rho3 | 2016 | IIc | 9 | CC9 | 1690 | 99.9 | SAMEA6856036 | PRJEB38495 |
| 16-LI00966-0 | Food | Rho3 | 2016 | IIc | 9 | CC9 | 1690 | 99.5 | SAMEA6856043 | PRJEB38495 |
| 16-LI00984-0 | Food | Rho3 | 2016 | IIc | 9 | CC9 | 1690 | 99.9 | SAMEA6856056 | PRJEB38495 |
| 16-LI00986-0 | Food | Rho3 | 2016 | IIc | 9 | CC9 | 1690 | 99.9 | SAMEA6856058 | PRJEB38495 |
| 16-LI01064-0 | Food | Rho3 | 2016 | IIa | 9 | CC9 | 1690 | 99.9 | SAMEA6856092 | PRJEB38495 |
| 16-LI01066-1 | Food | Rho3 | 2016 | IIc | 9 | CC9 | 1690 | 99.9 | SAMEA6856095 | PRJEB38495 |
| 16-LI01074-0 | Food | Rho3 | 2016 | IIc | 9 | CC9 | 1690 | 99.9 | SAMEA6856101 | PRJEB38495 |
| 16-LI01075-0 | Food | Rho3 | 2016 | IIc | 9 | CC9 | 1690 | 99.5 | SAMEA6856102 | PRJEB38495 |
| 16-LI01076-0 | Food | Rho3 | 2016 | IIc | 9 | CC9 | 1690 | 99.9 | SAMEA6856104 | PRJEB38495 |
| 16-LI01147-0 | Food | Rho3 | 2016 | IIc | 9 | CC9 | 1690 | 99.8 | SAMN22043219 | PRJNA768430 |
| 16-LI01158-0 | Food | Rho3 | 2016 | IIc | 9 | CC9 | 1690 | 99.9 | SAMEA6856146 | PRJEB38495 |
| 16-LI01165-0 | Food | Rho3 | 2016 | IIc | 9 | CC9 | 1690 | 99.5 | SAMEA6856152 | PRJEB38495 |
| 16-LI01201-0 | Food | Rho3 | 2016 | IIc | 9 | CC9 | 1690 | 99.9 | SAMEA6856172 | PRJEB38495 |
| 17-LI00008-0 | Food | Rho3 | 2016 | IIc | 9 | CC9 | 1690 | 99.9 | SAMEA6856186 | PRJEB38495 |
| 17-LI00071-0 | Food | Rho3 | 2017 | IIc | 9 | CC9 | 1690 | 99.9 | SAMN22043220 | PRJNA768430 |
| 17-LI00248-0 | Food | Rho3 | 2017 | IIc | 9 | CC9 | 1690 | 99.9 | SAMN22043221 | PRJNA768430 |
| 17-LI00327-0 | Food | Rho3 | 2017 | IIc | 9 | CC9 | 1690 | 99.5 | SAMN22043222 | PRJNA768430 |
| 17-LI00725-0 | Food | Rho3 | 2017 | IIc | 9 | CC9 | 1690 | 99.9 | SAMN22043223 | PRJNA768430 |
| 17-LI00959-0 | Food | Rho3 | 2017 | IIc | 9 | CC9 | 1690 | 99.9 | SAMN22043224 | PRJNA768430 |
| 17-LI01042-0 | Food | Rho3 | 2017 | IIc | 9 | CC9 | 1690 | 99.9 | SAMN22043225 | PRJNA768430 |
| 18-LI00028-0 | Food | Rho3 | 2017 | IIc | 9 | CC9 | 1690 | 99.9 | SAMN22043226 | PRJNA768430 |
| 18-LI00582-0 | Food | Rho3 | 2018 | IIc | 9 | CC9 | 1690 | 99.6 | SAMN22043227 | PRJNA768430 |
| 18-LI00901-0 | Food | Rho3 | 2017 | IIa | 9 | CC9 | 1690 | 99.3 | SAMN22043228 | PRJNA768430 |
| 18-LI00902-0 | Food | Rho3 | 2017 | IIa | 9 | CC9 | 1690 | 98.8 | SAMN22043229 | PRJNA768430 |
| 18-LI00905-0 | Food | Rho3 | 2017 | IIa | 9 | CC9 | 1690 | 99.7 | SAMN22043230 | PRJNA768430 |
| 18-LI00906-0 | Food | Rho3 | 2018 | IIa | 9 | CC9 | 1690 | 96.7 | SAMN22043231 | PRJNA768430 |
| 19-LI01228-0 | Food | Rho3 | 2019 | IIc | 9 | CC9 | 1690 | 99.9 | SAMN22043232 | PRJNA768430 |
| 20-LI01190-0 | Food | Rho3 | 2020 | IIc | 9 | CC9 | 1690 | 99.9 | SAMN22043233 | PRJNA768430 |
| 20-LI01519-0 | Food | Rho3 | 2020 | IIc | 9 | CC9 | 1690 | 99.8 | SAMN22043234 | PRJNA768430 |
| 20-LI01774-0 | Food | Rho3 | 2020 | IIc | 9 | CC9 | 1690 | 99.9 | SAMN22043235 | PRJNA768430 |
| 20-LI01850-0 | Food | Rho3 | 2020 | IIc | 9 | CC9 | 1690 | 99.8 | SAMN22043236 | PRJNA768430 |
| 18-06542 | clinical/host-associated | Rho8 | 2018 | IIa | 37 | CC37 | 7559 | 99.8 | SAMEA6800710 | PRJEB37942 |
| 20-00300 | clinical/host-associated | Rho8 | 2020 | IIa | 37 | CC37 | 7559 | 99.9 | SAMEA10017368 | PRJEB48063 |
| 20-00993 | clinical/host-associated | Rho8 | 2020 | IIa | 37 | CC37 | 7559 | 99.8 | SAMEA10017374 | PRJEB48063 |
| 20-01052 | clinical/host-associated | Rho8 | 2020 | IIa | 37 | CC37 | 7559 | 99.9 | SAMEA10017375 | PRJEB48063 |
| 20-01359 | clinical/host-associated | Rho8 | 2020 | IIa | 37 | CC37 | 7559 | 99.9 | SAMEA10018409 | PRJEB48063 |
| 20-01921 | clinical/host-associated | Rho8 | 2020 | IIa | 37 | CC37 | 7559 | 99.8 | SAMEA7540781 | PRJEB29295 |
| 20-04108 | clinical/host-associated | Rho8 | 2020 | IIa | 37 | CC37 | 7559 | 99.8 | SAMEA10017391 | PRJEB48063 |
| 20-04493 | clinical/host-associated | Rho8 | 2020 | IIa | 37 | CC37 | 7559 | 99.6 | SAMEA10017392 | PRJEB48063 |
| 21-02313 | clinical/host-associated | Rho8 | 2021 | IIa | 37 | CC37 | 7559 | 96.5 | SAMEA10017412 | PRJEB48063 |
| 17-LI00154-0 | Food | Rho8 | 2017 | IIa | 37 | CC37 | 7559 | 99.9 | SAMN22043237 | PRJNA768430 |
| 17-LI00922-0 | Food | Rho8 | 2017 | IIa | 37 | CC37 | 7559 | 99.9 | SAMN22043238 | PRJNA768430 |
| 19-LI01214-0 | Food | Rho8 | 2019 | IIa | 37 | CC37 | 7559 | 99.9 | SAMN22043239 | PRJNA768430 |
| 20-LI00563-0 | Food | Rho8 | 2020 | IIa | 37 | CC37 | 7559 | 99.8 | SAMN22043240 | PRJNA768430 |
| 20-LI00696-0 | Food | Rho8 | 2020 | IIa | 37 | CC37 | 7559 | 99.8 | SAMN22043241 | PRJNA768430 |
| 20-LI00699-0 | Food | Rho8 | 2020 | IIa | 37 | CC37 | 7559 | 99.8 | SAMN22043242 | PRJNA768430 |
| 20-LI01506-0 | Food | Rho8 | 2020 | IIa | 37 | CC37 | 7559 | 99.8 | SAMN22043243 | PRJNA768430 |
| 21-LI00189-0 | Food | Rho8 | 2021 | IIa | 37 | CC37 | 7559 | 99.8 | SAMN22043244 | PRJNA768430 |
| 21-LI00302-0 | Food | Rho8 | 2021 | IIa | 37 | CC37 | 7559 | 99.8 | SAMN22043245 | PRJNA768430 |
| 21-LI00890-0 | Food | Rho8 | 2021 | IIa | 37 | CC37 | 7559 | 99.8 | SAMN22043246 | PRJNA768430 |
| 18-04319 | clinical/host-associated | Sigma5 | 2018 | IIa | 504 | CC475 | 5715 | 99.4 | SAMEA6800701 | PRJEB37942 |
| 18-04417 | clinical/host-associated | Sigma5 | 2018 | IIa | 504 | CC475 | 5715 | 99.6 | SAMEA10017322 | PRJEB48063 |
| 20-02934 | clinical/host-associated | Sigma5 | 2020 | IIa | 504 | CC475 | 5715 | 99.2 | SAMEA10018417 | PRJEB48063 |
| 20-02961 | clinical/host-associated | Sigma5 | 2020 | IIa | 504 | CC475 | 5715 | 99.2 | SAMEA10018418 | PRJEB48063 |
| 20-04545 | clinical/host-associated | Sigma5 | 2020 | IIa | 504 | CC475 | 5715 | 99.2 | SAMEA10017393 | PRJEB48063 |
| 20-04588 | clinical/host-associated | Sigma5 | 2020 | IIa | 504 | CC475 | 5715 | 99.2 | SAMEA10017394 | PRJEB48063 |
| 20-05143 | clinical/host-associated | Sigma5 | 2020 | IIa | 504 | CC475 | 5715 | 99.2 | SAMEA10018419 | PRJEB48063 |
| 20-06674 | clinical/host-associated | Sigma5 | 2020 | IIa | 504 | CC475 | 5715 | 98.7 | SAMEA10017399 | PRJEB48063 |
| 21-00057 | clinical/host-associated | Sigma5 | 2021 | IIa | 504 | CC475 | 5715 | 97.8 | SAMEA10017400 | PRJEB48063 |
| 21-00268 | clinical/host-associated | Sigma5 | 2021 | IIa | 504 | CC475 | 5715 | 99.2 | SAMEA10017403 | PRJEB48063 |
| 16-LI00963-0 | Food | Sigma5 | 2016 | IIa | 504 | CC475 | 5715 | 99.6 | SAMEA6856040 | PRJEB38495 |
| 20-LI00698-0 | Food | Sigma5 | 2020 | IIa | 504 | CC475 | 5715 | 99.2 | SAMN22043247 | PRJNA768430 |
| 20-LI01874-0 | Food | Sigma5 | 2020 | IIa | 504 | CC475 | 5715 | 99.2 | SAMN22043248 | PRJNA768430 |
| 14-01878 | clinical/host-associated | Tau1a | 2014 | IIa | 155 | CC155 | 2198 | 99.5 | SAMEA10018372 | PRJEB48063 |
| 14-02713 | clinical/host-associated | Tau1a | 2014 | IIa | 155 | CC155 | 2198 | 99.5 | SAMEA10018373 | PRJEB48063 |
| 14-02773 | clinical/host-associated | Tau1a | 2014 | IIa | 155 | CC155 | 2198 | 97.3 | SAMEA10018374 | PRJEB48063 |
| 16-00804 | clinical/host-associated | Tau1a | 2016 | IIa | 155 | CC155 | 2198 | 99.5 | SAMEA104485240 | PRJEB24496 |
| 16-03142 | clinical/host-associated | Tau1a | 2016 | IIa | 155 | CC155 | 2198 | 99.5 | SAMEA104485345 | PRJEB24496 |
| 16-03191 | clinical/host-associated | Tau1a | 2016 | IIa | 155 | CC155 | 2198 | 99.5 | SAMEA104485348 | PRJEB24496 |
| 16-04140 | clinical/host-associated | Tau1a | 2016 | IIa | 155 | CC155 | 2198 | 99.5 | SAMEA104485392 | PRJEB24496 |
| 17-03594 | clinical/host-associated | Tau1a | 2017 | IIa | 155 | CC155 | 2198 | 99.5 | SAMEA10017301 | PRJEB48063 |
| 17-05800 | clinical/host-associated | Tau1a | 2017 | IIa | 155 | CC155 | 2198 | 97.3 | SAMEA10018392 | PRJEB48063 |
| 17-06202 | clinical/host-associated | Tau1a | 2017 | IIa | 155 | CC155 | 2198 | 99.3 | SAMEA10018395 | PRJEB48063 |
| 18-02988 | clinical/host-associated | Tau1a | 2018 | IIa | 155 | CC155 | 2198 | 99.3 | SAMEA10017312 | PRJEB48063 |
| 18-02989 | clinical/host-associated | Tau1a | 2018 | IIa | 155 | CC155 | 2198 | 99.6 | SAMEA10017313 | PRJEB48063 |
| 18-03122 | clinical/host-associated | Tau1a | 2018 | IIa | 155 | CC155 | 2198 | 99.6 | SAMEA6602610 | PRJEB29295 |
| 18-03123 | clinical/host-associated | Tau1a | 2018 | IIa | 155 | CC155 | 2198 | 99.5 | SAMEA6800714 | PRJEB37942 |
| 18-03210 | clinical/host-associated | Tau1a | 2018 | IIa | 155 | CC155 | 2198 | 99.6 | SAMEA10018400 | PRJEB48063 |
| 18-03741 | clinical/host-associated | Tau1a | 2018 | IIa | 2890 | CC155 | 2198 | 99.6 | SAMEA10017317 | PRJEB48063 |
| 18-04318 | clinical/host-associated | Tau1a | 2018 | IIa | 155 | CC155 | 2198 | 99.5 | SAMEA10017321 | PRJEB48063 |
| 18-05164 | clinical/host-associated | Tau1a | 2018 | IIa | 155 | CC155 | 2198 | 99.5 | SAMEA10017326 | PRJEB48063 |
| 18-05401 | clinical/host-associated | Tau1a | 2018 | IIa | 155 | CC155 | 2198 | 99.5 | SAMEA10017327 | PRJEB48063 |
| 18-06022 | clinical/host-associated | Tau1a | 2018 | IIa | 155 | CC155 | 2198 | 98.9 | SAMEA10018401 | PRJEB48063 |
| 18-06112 | clinical/host-associated | Tau1a | 2018 | IIa | 155 | CC155 | 2198 | 99.6 | SAMEA10018402 | PRJEB48063 |
| 18-06118 | clinical/host-associated | Tau1a | 2018 | IIa | ? | ? | 2198 | 95.2 | SAMEA10018424 | PRJEB48063 |
| 18-06120 | clinical/host-associated | Tau1a | 2018 | IIa | 155 | CC155 | 2198 | 99.5 | SAMEA10018425 | PRJEB48063 |
| 19-00224 | clinical/host-associated | Tau1a | 2019 | IIa | 2890 | CC155 | 2198 | 99.3 | SAMEA10017333 | PRJEB48063 |
| 19-00283 | clinical/host-associated | Tau1a | 2019 | IIa | 2890 | CC155 | 2198 | 99.6 | SAMEA10017334 | PRJEB48063 |
| 19-00284 | clinical/host-associated | Tau1a | 2019 | IIa | 155 | CC155 | 2198 | 99.6 | SAMEA10017335 | PRJEB48063 |
| 19-06645 | clinical/host-associated | Tau1a | 2019 | IIa | 155 | CC155 | 2198 | 98.5 | SAMEA10017355 | PRJEB48063 |
| 19-06729 | clinical/host-associated | Tau1a | 2019 | IIa | 155 | CC155 | 2198 | 97.5 | SAMEA10017357 | PRJEB48063 |
| 20-00038 | clinical/host-associated | Tau1a | 2019 | IIa | 155 | CC155 | 2198 | 99.6 | SAMEA10017365 | PRJEB48063 |
| 20-00733 | clinical/host-associated | Tau1a | 2020 | IIa | 155 | CC155 | 2198 | 99.6 | SAMEA10017373 | PRJEB48063 |
| 16-LI00786-0 | Food | Tau1a | 2016 | IIa | 155 | CC155 | 2198 | 99.6 | SAMEA6855934 | PRJEB38495 |
| 17-LI00881-0 | Food | Tau1a | 2017 | IIa | 155 | CC155 | 2198 | 99.6 | SAMN22043249 | PRJNA768430 |
| 18-LI00050-0 | Food | Tau1a | 2017 | IIa | 155 | CC155 | 2198 | 99.6 | SAMN22043250 | PRJNA768430 |
| 18-00275 | clinical/host-associated | Ypsilon3 | 2018 | IIa | 121 | CC121 | 5554 | 99.2 | SAMEA10018397 | PRJEB48063 |
| 18-00324 | clinical/host-associated | Ypsilon3 | 2018 | IIa | 121 | CC121 | 5554 | 99.8 | SAMEA6602611 | PRJEB29295 |
| 18-00325 | clinical/host-associated | Ypsilon3 | 2018 | IIa | 121 | CC121 | 5554 | 99.8 | SAMEA10018398 | PRJEB48063 |
| 16-LI00752-0 | Food | Ypsilon3 | 2016 | IIa | 121 | CC121 | 5554 | 99.9 | SAMEA6855919 | PRJEB38495 |
| 16-LI01062-0 | Food | Ypsilon3 | 2016 | IIa | 121 | CC121 | 5554 | 99.9 | SAMEA6856091 | PRJEB38495 |
| 16-LI01164-0 | Food | Ypsilon3 | 2016 | IIa | 121 | CC121 | 5554 | 99.9 | SAMEA6856151 | PRJEB38495 |
| 17-LI00336-0 | Food | Ypsilon3 | 2017 | IIa | 121 | CC121 | 5554 | 99.9 | SAMN22043251 | PRJNA768430 |
| 17-LI00869-0 | Food | Ypsilon3 | 2017 | IIa | 121 | CC121 | 5554 | 99.9 | SAMN22043252 | PRJNA768430 |
| 17-LI00904-0 | Food | Ypsilon3 | 2017 | IIa | 121 | CC121 | 5554 | 99.8 | SAMN22043253 | PRJNA768430 |
| 17-LI00905-0 | Food | Ypsilon3 | 2017 | IIa | 121 | CC121 | 5554 | 99.9 | SAMN22043254 | PRJNA768430 |
| 17-LI00909-0 | Food | Ypsilon3 | 2017 | IIa | 121 | CC121 | 5554 | 99.9 | SAMN22043255 | PRJNA768430 |
| 17-LI00911-0 | Food | Ypsilon3 | 2017 | IIa | 121 | CC121 | 5554 | 99.9 | SAMN22043256 | PRJNA768430 |
| 17-LI00951-0 | Food | Ypsilon3 | 2017 | IIa | 121 | CC121 | 5554 | 99.9 | SAMN22043257 | PRJNA768430 |
| 19-00348 | clinical/host-associated | Ypsilon6 | 2019 | IIa | 16 | CC8 | 3732 | 100.0 | SAMEA10018405 | PRJEB48063 |
| 19-01795 | clinical/host-associated | Ypsilon6 | 2019 | IIa | 16 | CC8 | 3732 | 99.6 | SAMEA10017336 | PRJEB48063 |
| 19-03248 | clinical/host-associated | Ypsilon6 | 2019 | IIa | 16 | CC8 | 3732 | 99.9 | SAMEA6602603 | PRJEB29295 |
| 19-06522 | clinical/host-associated | Ypsilon6 | 2019 | IIa | 16 | CC8 | 3732 | 99.8 | SAMEA10017353 | PRJEB48063 |
| 19-07077 | clinical/host-associated | Ypsilon6 | 2019 | IIa | 16 | CC8 | 3732 | 99.3 | SAMEA10017361 | PRJEB48063 |
| 20-02686 | clinical/host-associated | Ypsilon6 | 2020 | IIa | 16 | CC8 | 3732 | 99.7 | SAMEA10017386 | PRJEB48063 |
| 21-01684 | clinical/host-associated | Ypsilon6 | 2021 | IIa | 16 | CC8 | 3732 | 99.6 | SAMEA10017409 | PRJEB48063 |
| 21-02564 | clinical/host-associated | Ypsilon6 | 2021 | IIa | 16 | CC8 | 3732 | 99.2 | SAMEA10017414 | PRJEB48063 |
| 17-LI00483-0 | Food | Ypsilon6 | 2017 | IIa | 16 | CC8 | 3732 | 100.0 | SAMN22043258 | PRJNA768430 |
| 13-00745 | clinical/host-associated | Zeta1 | 2013 | IIa | 403 | CC403 | 40 | 99.6 | SAMEA104485076 | PRJEB24496 |
| 14-02065 | clinical/host-associated | Zeta1 | 2014 | IIa | 403 | CC403 | 40 | 99.6 | SAMEA104485093 | PRJEB24496 |
| 14-05969 | clinical/host-associated | Zeta1 | 2014 | IIa | 403 | CC403 | 40 | 99.6 | SAMEA104485115 | PRJEB24496 |
| 14-06239 | clinical/host-associated | Zeta1 | 2014 | IIa | 403 | CC403 | 40 | 99.6 | SAMEA104485117 | PRJEB24496 |
| 15-01123 | clinical/host-associated | Zeta1 | 2015 | IIa | 403 | CC403 | 40 | 99.7 | SAMEA104485141 | PRJEB24496 |
| 16-02860 | clinical/host-associated | Zeta1 | 2016 | IIa | 403 | CC403 | 40 | 99.6 | SAMEA104485323 | PRJEB24496 |
| 16-03014 | clinical/host-associated | Zeta1 | 2016 | IIa | 403 | CC403 | 3991 | 99.6 | SAMEA104485336 | PRJEB24496 |
| 16-03022 | clinical/host-associated | Zeta1 | 2016 | IIa | 403 | CC403 | 40 | 99.6 | SAMEA104485337 | PRJEB24496 |
| 16-03069 | clinical/host-associated | Zeta1 | 2016 | IIa | 403 | CC403 | 40 | 99.6 | SAMEA104485344 | PRJEB24496 |
| 16-03146 | clinical/host-associated | Zeta1 | 2016 | IIa | 403 | CC403 | 40 | 99.6 | SAMEA104485346 | PRJEB24496 |
| 16-03812 | clinical/host-associated | Zeta1 | 2016 | IIa | 403 | CC403 | 40 | 99.7 | SAMEA104485376 | PRJEB24496 |
| 16-04000 | clinical/host-associated | Zeta1 | 2016 | IIa | 403 | CC403 | 3991 | 99.6 | SAMEA104485386 | PRJEB24496 |
| 16-04624 | clinical/host-associated | Zeta1 | 2016 | IIa | 403 | CC403 | 40 | 99.6 | SAMEA104485415 | PRJEB24496 |
| 17-00225 | clinical/host-associated | Zeta1 | 2017 | IIa | 403 | CC403 | 40 | 99.7 | SAMEA104485447 | PRJEB24496 |
| 17-02249 | clinical/host-associated | Zeta1 | 2017 | IIa | 403 | CC403 | 40 | 99.6 | SAMEA10018386 | PRJEB48063 |
| 17-05468 | clinical/host-associated | Zeta1 | 2017 | IIa | 403 | CC403 | 6406 | 99.8 | SAMEA10018389 | PRJEB48063 |
| 20-03123 | clinical/host-associated | Zeta1 | 2020 | IIa | 403 | CC403 | 3991 | 99.2 | SAMEA10017387 | PRJEB48063 |
| 20-03231 | clinical/host-associated | Zeta1 | 2020 | IIa | 403 | CC403 | 3991 | 99.6 | SAMEA10017389 | PRJEB48063 |
| 21-03106 | clinical/host-associated | Zeta1 | 2021 | IIa | 403 | CC403 | 6406 | 99.4 | SAMEA10017416 | PRJEB48063 |
| 16-LI00812-0 | Food | Zeta1 | 2016 | IIa | 403 | CC403 | 40 | 99.8 | SAMEA6855948 | PRJEB38495 |
| 17-LI00164-0 | Food | Zeta1 | 2017 | IIa | 403 | CC403 | 40 | 99.8 | SAMN22043259 | PRJNA768430 |
| 17-LI00165-0 | Food | Zeta1 | 2017 | IIa | 403 | CC403 | 40 | 99.8 | SAMN22043260 | PRJNA768430 |
| 17-LI00901-0 | Food | Zeta1 | 2017 | IIa | 403 | CC403 | 40 | 99.7 | SAMN22043261 | PRJNA768430 |
| 18-02772 | clinical/host-associated | Zeta5a | 2018 | IVb | 6 | CC6 | 3386 | 99.4 | SAMEA10017310 | PRJEB48063 |
| 18-02900 | clinical/host-associated | Zeta5a | 2018 | IVb | 6 | CC6 | 3386 | 99.4 | SAMEA10017311 | PRJEB48063 |
| 18-03656 | clinical/host-associated | Zeta5a | 2018 | IVb | 6 | CC6 | 3386 | 99.5 | SAMEA6602604 | PRJEB29295 |
| 18-03918 | clinical/host-associated | Zeta5a | 2018 | IVb | 6 | CC6 | 3386 | 99.6 | SAMEA10017319 | PRJEB48063 |
| 18-04142 | clinical/host-associated | Zeta5a | 2018 | IVb | 6 | CC6 | 3386 | 99.1 | SAMEA10017320 | PRJEB48063 |
| 18-05101 | clinical/host-associated | Zeta5a | 2018 | IVb | 6 | CC6 | 3386 | 99.4 | SAMEA6800748 | PRJEB37942 |
| 18-06437 | clinical/host-associated | Zeta5a | 2018 | IVb | 6 | CC6 | 3386 | 99.6 | SAMEA10017329 | PRJEB48063 |
| 19-00083 | clinical/host-associated | Zeta5a | 2019 | IVb | 6 | CC6 | 3386 | 99.5 | SAMEA10017332 | PRJEB48063 |
| 19-00311 | clinical/host-associated | Zeta5a | 2019 | IVb | 6 | CC6 | 3386 | 99.6 | SAMEA10018404 | PRJEB48063 |
| 19-02962 | clinical/host-associated | Zeta5a | 2019 | IVb | 6 | CC6 | 3386 | 99.1 | SAMEA10018408 | PRJEB48063 |
| 18-LI00594-0 | Food | Zeta5a | 2018 | IVb | 6 | CC6 | 3386 | 99.6 | SAMN22043262 | PRJNA768430 |
| 18-LI00595-0 | Food | Zeta5a | 2018 | IVb | 6 | CC6 | 3386 | 99.6 | SAMN22043263 | PRJNA768430 |
| 18-LI00596-0 | Food | Zeta5a | 2018 | IVb | 6 | CC6 | 3386 | 99.6 | SAMN22043264 | PRJNA768430 |
| 19-LI00036-0 | Food | Zeta5a | 2018 | IVb | 6 | CC6 | 3386 | 97.8 | SAMN22043265 | PRJNA768430 |
| 19-LI00037-0 | Food | Zeta5a | 2018 | IVb | 6 | CC6 | 3386 | 99.6 | SAMN22043266 | PRJNA768430 |
| 19-LI00038-0 | Food | Zeta5a | 2018 | IVb | 6 | CC6 | 3386 | 99.6 | SAMN22043267 | PRJNA768430 |
| 19-LI00039-0 | Food | Zeta5a | 2018 | IVb | 6 | CC6 | 3386 | 99.6 | SAMN22043268 | PRJNA768430 |
| 19-LI00040-0 | Food | Zeta5a | 2018 | IVb | 6 | CC6 | 3386 | 99.5 | SAMN22043269 | PRJNA768430 |
| 19-LI00041-0 | Food | Zeta5a | 2018 | IVb | 6 | CC6 | 3386 | 99.5 | SAMN22043270 | PRJNA768430 |
| 19-LI00042-0 | Food | Zeta5a | 2018 | IVb | 6 | CC6 | 3386 | 99.5 | SAMN22043271 | PRJNA768430 |
| 19-LI00043-0 | Food | Zeta5a | 2018 | IVb | 6 | CC6 | 3386 | 99.6 | SAMN22043272 | PRJNA768430 |
| 19-LI00044-0 | Food | Zeta5a | 2018 | IVb | 6 | CC6 | 3386 | 99.6 | SAMN22043273 | PRJNA768430 |
| 19-LI00045-0 | Food | Zeta5a | 2018 | IVb | 6 | CC6 | 3386 | 99.6 | SAMN22043274 | PRJNA768430 |

Supplemental Table 2: **Characteristics and clonality parameters of the 22 outbreaks**

| **Cluster** | **serogroup** | **MLST ST** | **cgMLST CT** | **number of clinical isolates - Germany** | **allelic distance within clinical cluster min-max (median)** | **number of food isolates - Germany** | **allelic distance food vs clinical cluster min-max (median)** |
| --- | --- | --- | --- | --- | --- | --- | --- |
| Alpha4 | IIa | 8 | 1269 | 5 | 0-8 (5) | 2 | 0-9 (6) |
| Beta2a | IIa | 8 | 1247 | 13 | 1-12 (7) | 15 | 0-12 (7) |
| Chi1a | IIa | 14 | 2966, 5583 | 12 | 0-15 (7) | 9 | 0-14 (3) |
| Chi4 | IIa | 451 | 4035 | 2 | 1-1 (1) | 1 | 1-2 (1.5) |
| Chi6b | IVb | 6 | 1738, 9071 | 5 | 0-2 (0) | 1 | 6-8 (6) |
| Delta1 | IVb | 6 | 3530 | 22 | 0-4 (1) | 5 | 0-4 (1) |
| Delta8 | IIa | 121 | 4295 | 4 | 0-2 (1) | 2 | 4-9 (6.5) |
| Eta5 | IIa | 37 | 5488 | 7 | 0-4 (2) | 2 | 0-3 (1) |
| Eta8 | IIa | 37 | 4230 | 4 | 3-11 (8) | 2 | 2-8 (4.5) |
| Iota1c | IIa | 8 | 73, 1366, 6756 | 5 | 0-6 (3) | 1 | 1-6 (3) |
| My2 | IIa | 173 | 3242 | 19 | 0-12 (5) | 19 | 2-14 (7) |
| Omega5 | IIb | 87 | 773, 1138 | 11 | 0-10 (7) | 5 | 2-10 (6) |
| Omikron1 | IIa | 155 | 1128 | 42 | 0-13 (7) | 12 | 0-12 (5) |
| Omikron3 | IIa | 8 | 2994, 4997 | 8 | 0-7 (4) | 0 |  |
| Rho3 | IIa | 9 | 1690 | 11 | 0-11 (5) | 46 | 0-14 (6) |
| Rho8 | IIa | 37 | 7559 | 9 | 0-7 (3) | 10 | 0-6 (2) |
| Sigma5 | IIa | 504 | 5715 | 10 | 0-7 (3) | 3 | 1-6 (2.5) |
| Tau1a | IIa | 155 | 2198 | 30 | 0-16 (7) | 3 | 0-13 (4) |
| Ypsilon3 | IIa | 121 | 5554 | 3 | 0-0 (0) | 10 | 0-2 (1) |
| Ypsilon6 | IIa | 16 | 3732 | 8 | 0-3 (1) | 1 | 0-2 (2.5) |
| Zeta1 | IIa | 403 | 40, 3991, 6406 | 19 | 0-14 (3) | 4 | 0-10 (3) |
| Zeta5a | IVb | 6 | 3386, 6408 | 10 | 0-2 (0) | 13 | 0-4 (1) |
